# MAPS-seq: magnetic bead-assisted parallel single-cell gene expression profiling

**DOI:** 10.1038/s12276-020-0433-x

**Published:** 2020-05-13

**Authors:** Munsu Park, Dongin Lee, Duhee Bang, Ji Hyun Lee

**Affiliations:** 1 Department of Biomedical Science and Technology, Graduate School, Kyung Hee University, Seoul, Korea; 2 Department of Chemistry, Yonsei University, Seoul, Korea; 3 Department of Clinical Pharmacology and Therapeutics, College of Medicine, Kyung Hee University, Seoul, Korea; 4 Department of Biomedical Science and Technology, Kyung Hee Medical Science Research Institute, Kyung Hee University, Seoul, Korea

**Keywords:** Transcriptomics, Gene expression profiling, Transcriptomics

## Abstract

Recently developed single-cell RNA sequencing methods allow the simultaneous profiling of the transcriptomes of thousands of individual cells. However, current methods still require advanced equipment or entail substantial waste of reagents. Here, we introduce magnetic bead-assisted parallel single-cell gene expression sequencing (MAPS-seq), a microwell-based method that pools samples before the reverse transcription step, increasing the ease of sample preparation and reducing reagent waste. Moreover, because this method uses universal reagents and standard molecular biology lab instruments, it is easy to implement, even in labs that have not previously conducted single-cell RNA sequencing. We validated our method by demonstrating that it can generate gene expression data at the single-cell level. We then applied the MAPS-seq method to analyze 237 human myelogenous leukemia cells treated with one of three different drugs or dimethyl sulfoxide. We observed transcriptional changes and identified marker genes that indicate a drug response. Furthermore, the MAPS-seq method produced data of comparable quality to those of existing single-cell RNA sequencing methods. Consequently, we expect that our method will provide researchers with a more accessible, less wasteful, and less burdensome method for investigating the transcriptomes of individual cells.

## Single-cell genetic profiling: Promising magnetic method

A magnetically assisted gene profiling technique provides a less wasteful and laborious route to single-cell RNA sequencing. Ji Hyun Lee, Duhee Bang and co-workers at Kyung Hee and Yonsei universities in Seoul, South Korea, have developed ‘magnetic bead-assisted parallel single-cell gene expression sequencing’ (MAPS-seq) as an alternative to existing methods where individual cell samples must all be processed separately. By attaching cells to magnetic beads coated in ‘barcode’ marker molecules, MAPS-seq improves the efficiency of cell washing and allows the uniquely marked samples to be pooled in a single tube, saving on laboratory reagents. The researchers demonstrated MAPS-seq by separating human and mouse cells, and identifying genetic responses to drugs in leukemia cells. The technique potentially has numerous clinical and research applications, such as separating small numbers of circulating tumor cells from cancer-patient blood samples.

## Introduction

Organisms contain various types of cells with distinct cellular functions^1^. Given that the transcriptional state of a cell is closely related to its cellular function, cells can be investigated by profiling their transcriptomes. Specifically, cellular identity can be established with enhanced resolution by profiling the transcriptome of individual cells^2–6^. Research on single-cell transcripts has recently and rapidly expanded in various fields, such as the study of developmental processes in heterologous tissues of plants and the study of rare subspecies of cancer cells^7,8^.

In particular, as the need for single-cell research has become more important, various single-cell RNA sequencing (scRNA-seq) methods have been developed^9^. Representative scRNA-seq library generation methods include microwell-based methods, such as CEL-Seq and MARS-Seq^10,11^, and microfluidic platforms, such as Drop-seq, inDrop, and 10x genomics^12–15^. In microwell-based methods, the transcripts of cells are separated into microwells and individually reverse transcribed. However, these methods require a significant amount of labor and reagents because the samples must be processed separately before they are pooled. Microfluidic platforms are based on the separation of individual cells using nanoliter-sized droplets. However, this process requires complex equipment with high setup costs^16^.

In this work, we developed a new method, magnetic bead-assisted single-cell gene expression sequencing (MAPS-seq), which can be used to simultaneously analyze hundreds of cells with greater convenience and at lower cost than those of existing scRNA-seq methods. A comparison of MAPS-seq with representative scRNA-seq methods is presented in Table 1. We used streptavidin-coated magnetic beads bound with biotinylated cell-specific primers to pool samples at an early stage and applied the beads’ magnetism to increase the efficiency of the washing process. We validated our method by separating human and mouse cell lines. Furthermore, we demonstrated that our method can be applied to drug response studies. Using MAPS-seq, we observed transcriptional changes in the drug-treated K562 cells and detected expression markers that indicate a drug response. This convenient new method expands the ability of microwell-based methods and will vitalize diverse areas of biological study related to cellular transcription.

**Table 1:** Comparison of MAPS-seq with other representative scRNA-seq methods.

Method	CEL-Seq2	MARS-Seq2	Drop-seq	MAPS-seq
Region	3′ end	3′ end	3′ end	3′ end
Cell isolation	Manual	FACS	Microfluid	FACS
Cell barcode	Yes	Yes	Yes	Yes
UMI	Yes	Yes	Yes	Yes
Sample pooling	After cDNA amplification	After RT	Before RT	Before RT
Amplification	IVT	IVT	PCR	PCR
Fragmentation	Chemical	Chemical	Tn5 transposase	Tn5 transposase
Feature	Linear amplification	Automated massively parallel scRNA-seq	Nanoliter-sized droplet based high-throughput microfluidic equipment	Using magnetic beads for sample pooling

*FACS* fluorescence-activated cell sorting, *UMI* unique molecular identifier, *RT* reverse transcription, *IVT* in vitro transcription.

## Materials and methods

### Cell lines and cell culture

All cell lines were obtained from the Korean Cell Line Bank and maintained at 37 °C with 5% CO_2_. The human embryonic kidney 293T (HEK293T) cell line and the mouse embryo fibroblast NIH/3T3 cell line were cultured in Dulbecco’s modified Eagle’s medium (Gibco, USA) supplemented with 10% fetal bovine serum (FBS; Gibco, USA) and 1% penicillin/streptomycin (Thermo Fisher Scientific, USA). The human chronic myelogenous leukemia K562 cell line was cultured in Roswell Park Memorial Institute medium (Gibco, USA) supplemented with 10% FBS and 1% penicillin/streptomycin.

### Sequence of biotinylated cell-specific barcode oligos

We designed biotinylated cell-specific barcode oligos (BCOs) as follows: 5′-/biotin/AGTGGTATCAACGCAGAGTAC/JJJJJJ/NNNNNNN/(T)_26_-3′. Each oligo contains one biotin molecule on its 5′ end, followed by a SMART PCR primer^17,18^ binding site with the sequence AGTGGTATCAACGCAGAGTAC (Supplementary Table 1). JJJJJJ represents a 6-bp cell-specific barcode, and NNNNNNN represents a 7-bp unique molecular identifier (UMI) for each mRNA in an individual cell. Next, there is a polythymidine tail (T)_26_, which captures the poly-A tail of mRNA and is the start site of reverse transcription (Integrated DNA Technologies, USA) (Supplementary Fig. 1, Supplementary Table 2).

### Procedure of MAPS-seq

Streptavidin C1 beads (Invitrogen, USA) were washed and added to a 96-well plate (10 μg per well). BCOs were added to each well, resulting in the formation of BCO-conjugated streptavidin beads. The procedure for washing and combining the beads with BCOs was conducted according to the manufacturer’s instructions.

Four microliters of cell lysis buffer (10 mM Tris-HCl, pH 7.4; 10 mM NaCl; 3 mM MgCl_2_, and 0.1% IGEPAL CA-630)^19^ was added to each well of a new 96-well plate. Then, one cell was added to each well using an Aria II fluorescence-activated cell sorting (FACS) sorter (BD Biosciences, USA). The first was gated using the forward scatter (FSC) area vs. the side scatter (SSC) area (FSC-A vs. SSC-A) to remove dead cells or debris from the sample. The doublet was then removed using the FSC height vs. the FSC width (FSC-H vs. FSC-W) and the SSC height vs. the SSC width (SSC-H vs. SSC-W). After cell sorting, the plate was briefly centrifuged at 4 °C to allow the cells to sink into the lysis solution.

BCO-conjugated streptavidin beads were added to each cell well, adjusting the final volume of each well to 10 μL. The 96-well plate was then incubated at 55 °C for 5 min to allow the BCOs to capture mRNAs, and the plate was immediately placed on ice for at least 1 min. The beads were immobilized on the magnetic stand, and supernatants were removed. The beads were then washed twice and resuspended in ice-cold 6× saline-sodium citrate (SSC) buffer. All beads from the 96-well plate were then pooled into a microtube and washed once again with ice-cold 6× SSC buffer.

A reverse transcriptase (RTase) mixture was prepared with the following composition and added to the pooled beads (50 μL of mixture per microtube): 20 μL of nuclease-free water (Invitrogen, USA), 10 μL of 20% Ficoll PM400, 10 μL of 5× Maxima RT buffer, 5 μL of 10 mM dNTPs, 1.25 μL of RNase inhibitor, 1.25 μL of 100 μM template-switching oligo (TSO), and 2.5 μL of Maxima H Minus RTase (Thermo Scientific, USA). Reverse transcription was performed at 25 °C for 30 min. Then, the beads were washed twice with TE-TW buffer (10 mM Tris pH 8.0, 1 mM EDTA, and 0.01% Tween-20) and once with 10 mM Tris pH 8.0.

Exonuclease I mixture was prepared with the following composition and added to the pooled beads (50 μL of mixture per microtube): 42.5 μL of nuclease-free water, 5 μL of 10× Exo I buffer, and 2.5 μL of Exo I nuclease (NEB, USA). After incubation at 37 °C for 45 min, the beads were washed twice with TE-TW buffer and once with nuclease-free water.

The PCR mixture was prepared as follows: 24.6 μL of nuclease-free water, 0.4 μL of SMART primer (Integrated DNA Technologies, USA), and 25 μL of 2× KAPA HiFi HotStart ReadyMix (Roche, Switzerland). Then, 120 μg of beads was aliquoted into PCR tubes, and 50 μL of the PCR mixture was added to each tube, completely resuspending the beads in the PCR reaction mixture. PCR amplification was carried out using the following thermal cycler program: 3 min at 95 °C; 4 cycles of (20 s at 98 °C, 45 s at 60 °C, 3 min at 72 °C); 12-15 cycles of (20 s at 98 °C, 20 s at 67 °C, 3 min at 72 °C); and 5 min at 72 °C. The PCR products were double-purified with 0.6× AMPure XP beads (Beckman Coulter, USA) according to the manufacturer’s instructions.

A Nextera XT DNA library preparation kit (Illumina, USA) was used for transposition and library amplification. In that process, 0.6 ng of cDNA per sample was used. Then, the amplified libraries were purified, quantified, and sequenced on an Illumina NextSeq 500/550 system using custom procedures: read 1 was 20 bp (1–6 bases of cell barcode and 7–13 bases of UMI); read 2 was 50 bp to a paired end.

### Drug screening experiment

K562 human myelogenous leukemia cells were dispensed into a six-well plate (TPP, Switzerland) at 30% confluency and treated with 1 μM imatinib, rapamycin, or vinorelbine. A control sample was treated with 1 μM dimethyl sulfoxide (DMSO). After 48 h, the drug-treated cells and the control sample were analyzed by MAPS-seq.

### Single-cell transcriptome data processing

Demultiplexing, trimming, alignment, and annotation were performed according to the modified Drop-seq pipeline (http://mccarrollab.com/dropseq)^13^. Briefly, reads in the standard Drop-seq pipeline were modified by tagging according to the 6-bp cell-specific barcode sequence and the 7-bp UMI found in the 20-bp sequence from the 3′ end of “Read 1”. Then, “Read 2”, the paired end, was aligned with the hg19 (drug screening experiment) or hg19-mm10 (the other experiment) concatenated reference sequence, depending on the experiments, and collapsed onto 6-bp cell barcodes that corresponded to individual cells. A Hamming distance of 1 was used to collapse UMIs within each transcript and to collapse cell-specific barcodes within each cell. A digital expression matrix was obtained by collapsing the filtered and mapped reads for each gene by the UMI sequence within each cell barcode^20^.

### Analysis of drug screening data

After obtaining a digital expression matrix for the single-cell drug screening experiment, the following analysis was performed using the R package Seurat^21^. The data for each cell were quality controlled based on the mitochondrial read ratio and the number of genes. The mitochondrial read ratio is the ratio of mitochondrial reads to the total reads in each cell. Cells were kept if the number of genes exceeded 500 (the upper boundary was 6000–10,000 depending on the experiment) and if the mitochondrial read ratio was below 0.05. Principal component analysis (PCA) was performed using a variable gene, and t-distributed stochastic neighbor embedding (t-SNE)^22^ was performed to visualize the clusters.

After performing PCA and t-SNE using principal components from 1 to 4, we used Seurat’s FindCluster function^21^ to identify six clusters with a resolution of 1.5. For generation of a hierarchical cluster heatmap, the *p* value of a Wilcoxon rank-sum test was adjusted to 0.05, and the expression for each drug was confirmed. A total of 262 genes were identified by the expression of the three drugs (Supplementary Table 3). To determine the cell cycle phase of each cell^23^, we assigned cell cycle phase scores using cell cycle markers and classified each cell as G2/M, S, or G1 phase.

Before comparing our data set with the Drop-seq data set, we removed the batch effect that can arise between data produced by two different methods using the removeBatchEffect function in the R package edgeR^24^. The average expression level for each drug in each cell was calculated from the datasets created by each method. The expression level of each drug was calculated as log_2_[counts per million (CPM) + 1].

### Comparison of data trends between MAPS-seq and Drop-seq

We downloaded Drop-seq single-cell RNA sequencing data from NCBI GEO (accession GSE63473) to use as a human and mouse mixed species data set for comparison to our mixed species MAPS-seq data. To compare single-cell gene and transcript numbers between the Drop-seq data and the MAPS-seq data, we selected three representative cells (one human, one mouse, and one unidentified). We then compared the numbers of genes, transcripts, and read counts and the ratio of transcripts to read counts among the cells having at least 5000 transcripts from each data set.

## Results

### Overview of MAPS-seq

**Fig. 1:**
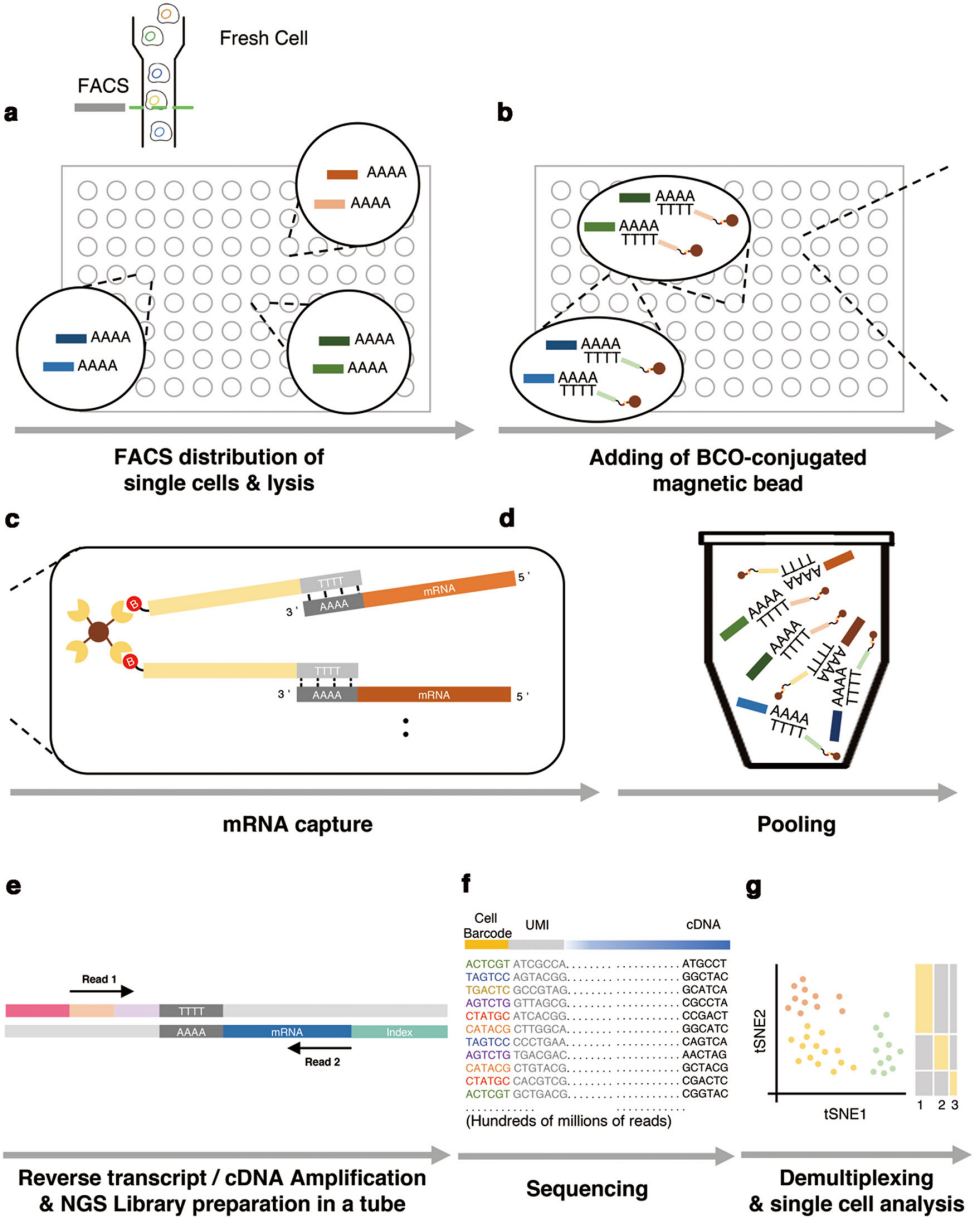
Principle and workflow of MAPS-seq. **a** Cells were dispensed into 96-well plates using a FACS sorter. **b** The binding reaction of streptavidin beads with biotin forms strong noncovalent bonds. Streptavidin beads conjugated with biotinylated cell-specific barcode oligos (BCOs) are added to each well. **c** Streptavidin beads conjugated with BCOs capture the poly-A sequence of mRNA transcripts. **d** All wells are pooled into a single tube. **e** Reverse transcription, cDNA amplification, and NGS library preparation were performed as in the standard Drop-seq method. **f** Sequencing was conducted using an Illumina 75 bp NGS system. “Read 1” contains information about the cell barcode and UMI; “Read 2” contains information about the transcript. **g** Data are demultiplexed and assigned to the originating cell. After postprocessing, these data can be expressed in various ways.

MAPS-seq consists of the following steps (Fig. 1): (i) Cells were distributed to a 96-well plate, one cell per well, using FACS. The cells were lysed by predispensed cell lysis buffer in each well. (ii) The BCO-conjugated beads were transferred to wells containing lysed cells. Each well (and thus each cell) received beads conjugated with BCOs containing a different cell-specific barcode. The BCOs then captured the mRNAs in each well, labeling them with a cell-specific barcode and UMI. This labeling assigned each transcript a unique barcode and UMI combination, making it possible to pool all the wells into a single tube. (iii) All BCO-conjugated streptavidin beads from one 96-well plate were pooled. This pooling is much more efficient than previous microwell-based methods because it reduces the number of samples handled in later steps from 96 to 1 (ref. ^11^). Reverse transcription (RT) was used to synthesize first-strand cDNA from each mRNA and extend its 3′ end with a complementary sequence of TSO. To efficiently amplify cDNA, we chose PCR over in vitro transcription (IVT). Although both methods are commonly used for RNA amplification, in a previous study comparing those two methods, IVT produced a relatively small amount of product, whereas PCR showed greater reproducibility among replicates^25^. Therefore, the PCR method is a better option when working with small starting amounts of RNA. In addition, a magnet was used to fix the beads, facilitating the washing process to remove unwanted residual reagents remaining after RT. (iv) The template-switching reaction proceeds to PCR. Only one primer is required; the single primer is complementary to the TSO sequence introduced at the 3′ end of cDNA and to the matching BCO sequence introduced at the 5′ end of the cDNA during RT^17,18^. Although we started with small amounts of mRNA, amplification by PCR produced sufficient cDNA for sequencing. (v) After PCR amplification, the cDNA libraries were subjected to transposition with transposon 5 (Tn5) transposase, which cleaves cDNA and adds specific sequences, making it easy to create NGS libraries. A final PCR amplification was then performed to prepare for massively parallel sequencing. (vi) Amplicons were sequenced by NGS, and the 3′ end sequences of the mRNAs were produced. These 3′ end sequences were transformed into a digital gene expression (DGE) matrix, which contains all cell-specific gene expression numbers. Further analysis was then performed using this matrix.

### Validation of MAPS-seq

**Fig. 2:**
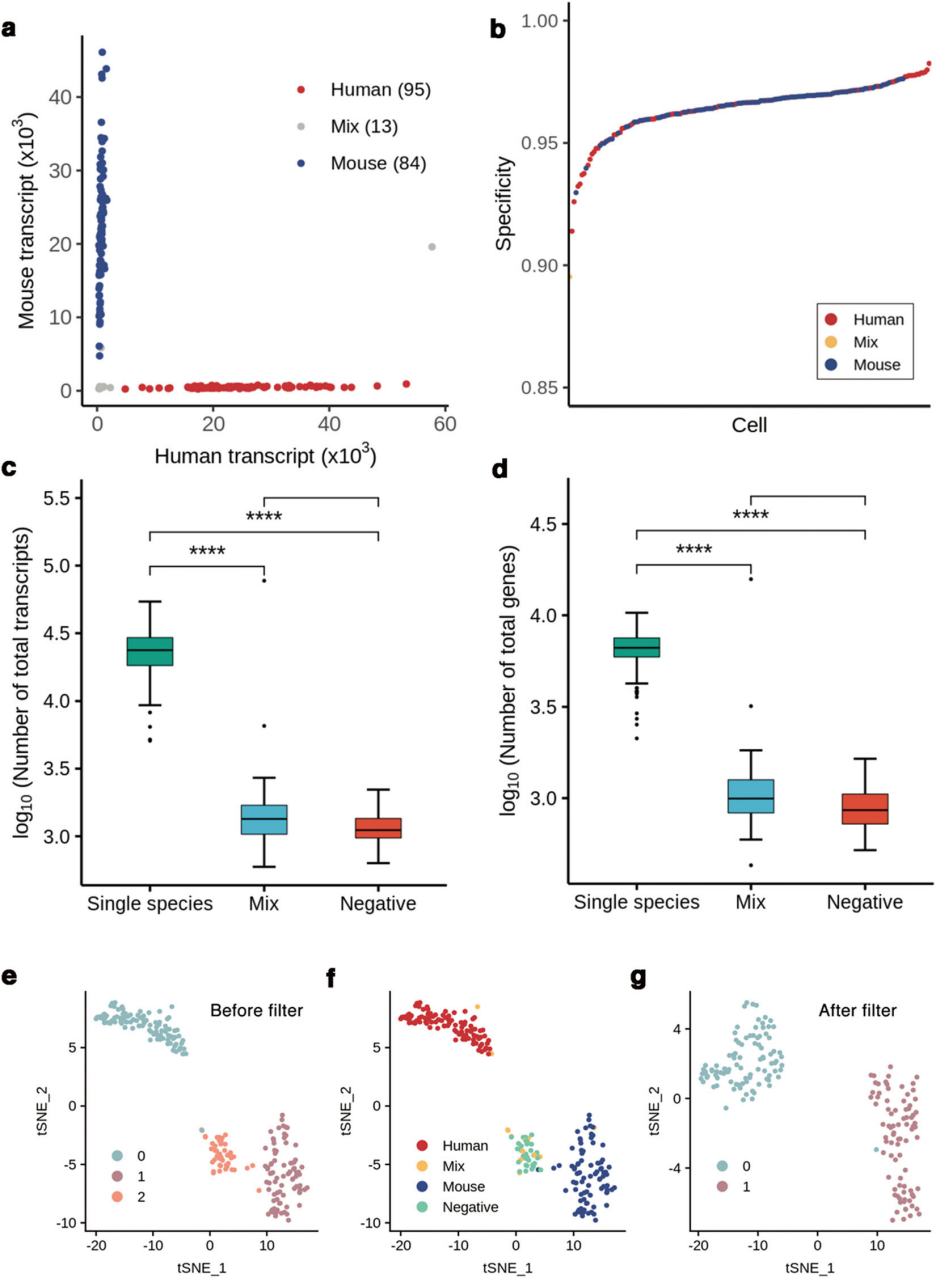
Species-mixing experiment using MAPS-seq. **a** The species-mixing experiment used 96 HEK293T and 96 NIH/3T3 cells, and the species of each cell was identified using its specificity. **b** Plot of the specificity values for each cell (180 cells), determined after quality control for the transcripts. The *x*-axis represents each cell, and the *y*-axis represents specificity. **c, d** Boxplots showing the total number of transcripts (**c**) and genes (**d**) found in the species-mixing experiment. Single species: human and mouse samples, identified by the specificity of each cell. Unidentified: mixed species identified by the specificity of each cell. Negative: control samples obtained by inserting empty beads. Each *y*-axis value is transformed to log_10_. Significance (****) is indicated when *p* ≤ 0.0001. **e** t-SNE visualization of single cells identified by performing unsupervised clustering analysis using digital gene expression (DGE) data before sample filtering. Cluster names were specified by random numbers. **f** Overlay of the original information about the sample in **e**. **g** t-SNE visualization performed after removing the low-quality sample.

To assess the performance of MAPS-seq, we tested the method with 96 HEK293T cells and 96 NIH/3T3 cells. To determine whether the cells were properly inserted into each well by FACS and captured by BCOs, we added 32 negative control wells where cells were not added. After sequencing of the library, we obtained a mean of 6.0 × 10^4^ reads per barcode for the 192 cells, excluding the negative controls. We removed cells with low or abnormally high numbers of transcripts so that the number of transcripts per cell was between 5 × 10^3^ and 6 × 10^4^. This process left 180 of 192 cells. Ninety-five of the 96 HEK293T cells and 84 of the 96 NIH/3T3 cells were clearly separated from their designated species (Fig. 2a). Among these cells, 162 cells had a human or mouse specificity over 0.95 (Fig. 2b). The human specificity was determined as the ratio of the number of human transcripts to the total number of transcripts. The mouse specificity was obtained in the same way. If the human specificity was greater than 0.9, the cells were identified as human cells. Conversely, if the mouse specificity was greater than 0.9, the cells were identified as mouse cells. Cells that did not meet these criteria were labeled unidentified. On average, per cell, the cells identified as a single species contained 6.3 × 10^3^ genes and 2.5 × 10^4^ transcripts, the negative controls contained 8.9 × 10^2^ genes and 1.1 × 10^3^ transcripts, and the unidentified cells contained 2.2 × 10^3^ genes and 7.5 × 10^3^ transcripts (Fig. 2c, d). One of the unidentified cells had an abnormally high number of transcripts, which we assumed to be the result of that cell actually being a heterogeneous doublet. Except for the presumed doublet, the unidentified cells contained 9.6 × 10^2^ genes and 1.3 × 10^3^ transcripts per cell on average, which were similar to those of the negative controls. We introduced a negative control to examine the possibility of false outcomes due to ambient RNA produced by transient DNA:RNA dissociation during the pooling process. As a result, we detected a small amount of ambient RNA, which was approximately 4.86% of the mean number of transcripts per cell.

To compare the MAPS-seq data with data produced by another single-cell RNA sequencing method, we used Drop-seq data from human and mouse cells. The pattern of transcript numbers for all the genes from selected representative human, mouse, and unidentified cells was similar between the two sequencing methods (Supplementary Fig. 2a–d). Additionally, the gene, transcript, and read count numbers from selected representative human, mouse, and unidentified cells were similar between the two sequencing methods. The read count numbers, however, were approximately three times higher in the Drop-seq data than in the MAPS-seq data, but the ratio of read counts to transcripts per cell was 0.15 for Drop-seq and 0.40 for MAPS-seq. MAPS-seq could detect more genes than Drop-seq because MAPS-seq had a relatively smaller amplification bias, so there were not many overlapping UMIs (Supplementary Fig. 2e–i).

Next, we performed PCA and t-SNE on the MAPS-seq transcriptome data from all 224 wells. By performing unbiased clustering, we could observe three clusters (Fig. 2e). When we labeled each cell with its original information, it was confirmed that the three clusters represented human, mouse, and negative control cells (Fig. 2f). Most of the cells that were removed by transcript-number filtering were negative control cells (Fig. 2g). Twelve of the cells removed with the negative controls were identified as mixed. We assumed that these wells did not actually contain any cells because of FACS errors and became contaminated by minute amounts of mRNA during the pooled RT step, which was bound by the BCOs after the pooling step, resulting in false-positive results. Our results demonstrated that it is possible to eliminate false results due to technical errors by using transcript-number filters.

Overall, we demonstrated that MAPS-seq performed well, leading to clear separation by species, despite the pooling of the cells. We also showed that data resulting from FACS errors can be easily removed with gene number filtering, thus preserving the overall data quality.

### Optimization of MAPS-seq

To enhance the performance of MAPS-seq, we sought to optimize the method with various experimental conditions. We first sought to optimize the initial amount of streptavidin beads dispensed to each cell to maximize the acquisition of cell information while reducing bead waste. We tested four different amounts of streptavidin beads (5, 10, 20, and 25 μg per cell) and used a total of 16 cells (8 HEK293T cells and 8 NIH/3T3 cells) for each condition. We obtained a mean of 5.8 × 10^4^ reads per cell and observed that when 10, 20, and 25 μg of streptavidin beads were used per cell, the mean number of transcripts per cell was similar (2.3 × 10^4^, 2.7 × 10^4^, and 2.4 × 10^4^, respectively) (Supplementary Fig. 3a). However, the number of transcripts and genes severely decreased when 5 μg of streptavidin beads was used per cell (mean 9.1 × 10^3^ transcripts and 3.2 × 10^3^ genes) (Supplementary Fig. 3a). The Wilcoxon rank-sum test revealed no significant differences between each pair of conditions among the three higher amounts (10, 20, and 25 μg), but there was a significant difference in the number of transcripts generated between the 5 μg condition and the other conditions (*p* value < 0.0001). This result confirmed that as the amount of streptavidin bead increases, the amount of data obtained increases, but there is no improvement for amounts of beads greater than 10 μg.

We also investigated the species specificity of each condition. With 5 µg of streptavidin beads per cell, two severely deviated cells were observed (Supplementary Fig. 3b). In contrast, when we used more than 10 µg per cell, the specificity values were uniform. As a result, at least 10 μg of streptavidin beads is required to effectively capture the mRNAs. When examining data from samples in which 20 or 25 μg of streptavidin beads was used, we did not observe any significant enhancement of the data quality compared to that of the 10 μg data. Consequently, we used 10 μg of streptavidin beads per cell for the most efficient use of the beads.

Next, we investigated the possibility that a relatively excessive amount of streptavidin beads in one PCR could hamper the PCR amplification of reverse-transcribed templates. To determine the optimal amounts of beads, we experimented with the same amount of template and different amounts of beads in each 50 μL PCR mixture. To confirm the amplification in the absence of beads, we amplified a control sample without beads. After PCR amplification, amplicons of each condition were quantified individually. We then compared the gene and transcript information obtained from each condition. As expected, the amplicon concentration was high (>50 ng/μL) when no beads were present during the PCR. As the amount of streptavidin beads gradually increased, the amplicon concentration decreased because the beads inhibited the PCR reaction. When the streptavidin bead amount was 200 µg per PCR, the amplicon concentration was 2.5 ng/μL (Supplementary Table 4). However, when comparing the correlations of gene expression between the condition without the beads and the conditions with the beads, the Pearson correlation coefficient was greater than 0.95 for all conditions (Supplementary Fig. 3c), showing that there was no significant difference in the data among the different conditions. In addition, when beads were present, we observed 2.3 × 10^4^ genes and 9.6 × 10^5^ transcripts on average, regardless of how many beads were used (Supplementary Fig. 3d). In summary, although the PCR amplification process was slightly inhibited by the streptavidin beads, the amount of streptavidin beads per PCR mixture had little impact on the outcome of our method within the investigated range.

### Analysis of drug-treated cells using MAPS-seq

**Fig. 3:**
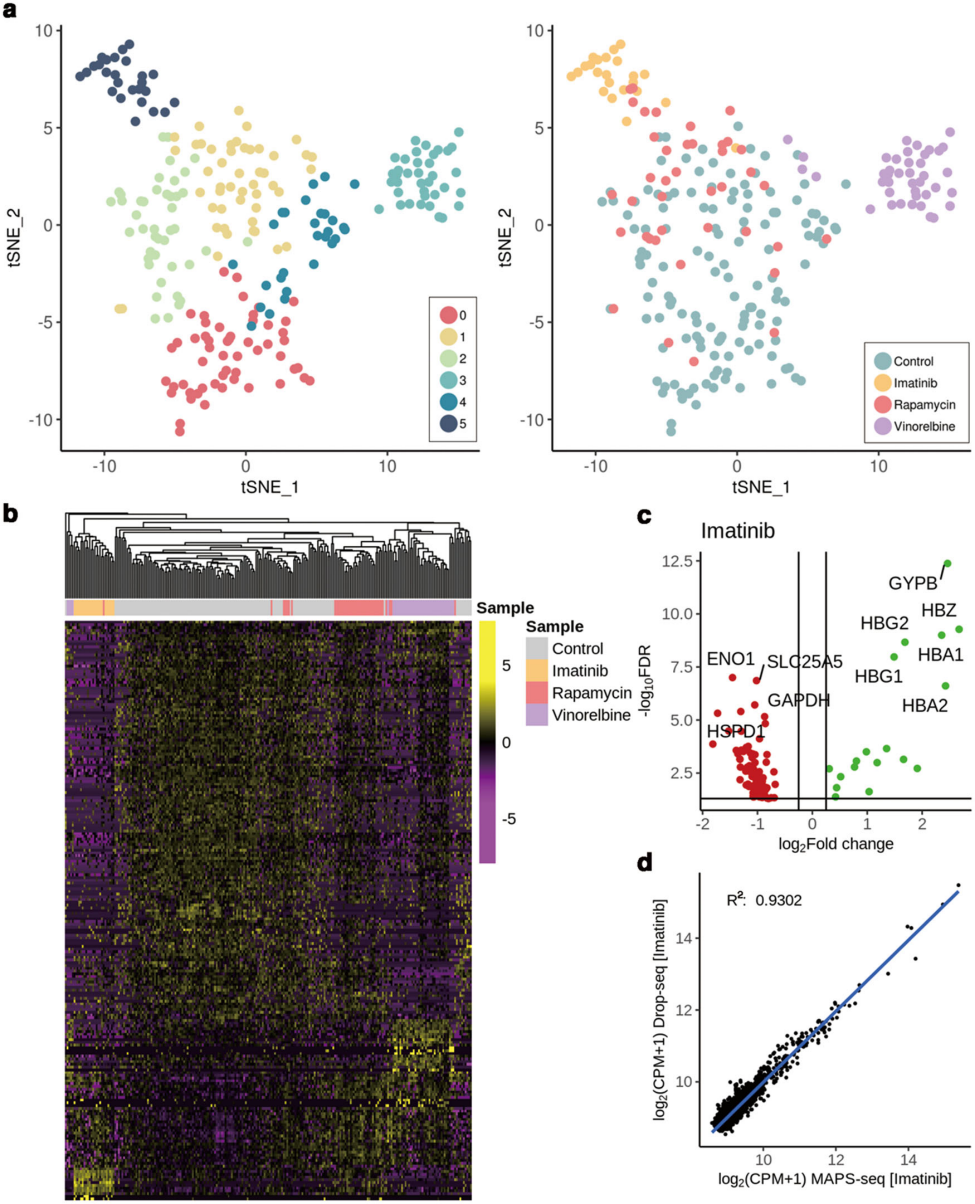
Gene expression analysis in drug treatment experiments. **a** t-SNE visualization of single cells identified by performing unsupervised clustering analysis using DGE data in drug treatment experiments via MAPS-seq (left). Clusters were numbered randomly. The original information about the sample is overlaid on the cluster analysis in the right panel. **b** Hierarchically clustered heatmap of gene expression profiles for drug treatment experiments in each K562 cell. Each column represents the expression value from the drug, and each row represents a gene. The heatmap is constructed from differentially expressed genes. **c** Volcano plot displaying differentially expressed genes of the imatinib-treated cells compared with the DMSO-treated controls. Genes with a *p* value <0.05 and an absolute value of log_2_ fold change (FC) > 0.25 are considered significant. Upregulated genes are green, downregulated genes are red, and noncritical genes are gray. The ten genes with the lowest *p* values are labeled. The cutoff for upregulation was log_2_ FC > 0.25 and the false discovery rate (FDR) was <0.05. The cutoff for downregulation was log_2_ FC < −0.25 and the FDR was <0.05. **d** Gene expression correlation for imatinib between MAPS-seq and Drop-seq.

The transcriptional response of a cell to a drug can be powerful evidence to uncover the action of the drug^26^. To support the wide applicability of our method, we sought to demonstrate that MAPS-seq can be applied to single-cell drug screening analysis. In previous studies, we identified drugs that cause pronounced changes in gene expression and used them to determine whether MAPS-seq can detect those transcriptional changes^27^. In the present study, we treated K562 leukemia cells (which contain a BCR-ABL fusion gene) with one of four treatments: imatinib, an antileukemia drug that targets BCR-ABL; rapamycin, an antitumor drug that does not specifically target BCR-ABL; vinorelbine, another antitumor drug that does not target BCR-ABL; or DMSO. We performed MAPS-seq with 48 cells treated with each drug and 144 DMSO-treated cells. After the data were filtered based on gene number and the mitochondrial read ratio, data remained for 237 cells, which represented 82% of the sorted cells (Supplementary Fig. 4a). We performed PCA and t-SNE analysis on the data for these 237 cells. The cells that were treated with imatinib or vinorelbine formed separate clusters, while the rapamycin-treated and DMSO-treated cells were commingled in one cluster (Fig. 3a). In addition, the G1 phase was dominant in a large proportion of the imatinib-treated cells (Supplementary Fig. 4a), which is concordant with a previous study^28^. Imatinib inhibits cell proliferation and induces cell cycle arrest in the G0/G1 phase through inactivation of extracellular signal-regulating kinases and blocking of the AKT signaling pathway. Differentially expressed genes were observed in each pair of drug conditions (Fig. 3b).

We next investigated the differentially expressed genes for each drug group compared with the DMSO group. We identified significant genes using the *p* value of the Wilcoxon rank-sum test: a *p* value less than 0.05 indicated that a gene is significant. In the imatinib-treated cells, we observed that 18 genes were upregulated, and 92 genes were downregulated [false discovery rate (FDR) < 10^−0.05^]. The most significantly differentially expressed genes were related to hemoglobin, such as *HBA*, *HBG*, and *HBZ* (upregulated) (Fig. 3c). The cells treated with rapamycin, an inhibitor of mTOR kinase, exhibited increased expression of *RPL* and *RPS*, which encode ribosomal proteins, and suppressed expression of *DDIT4*, which is involved in the mTOR signaling pathway (FDR < 10^−^^0.05^; Supplementary Fig. 4b)^29^. Vinorelbine acts as an antitumor drug by inhibiting mitosis through interaction with tubulin^30^. Consistent with its mechanism, vinorelbine was associated with the downregulation of tubulin-related genes in K562 cells, such as *TUBA*, *TUBB*, and *BEX4* (FDR < 10^−0.05^, Supplementary Fig. 4b). These results confirmed that the MAPS-seq transcriptome profiling data for each drug were consistent with previously reported drug mechanisms.

We also evaluated the reproducibility of our results by comparing the MAPS-seq data for single-cell drug screening analysis to the K562 drug screening data analyzed in our previous study using Drop-seq^27^. A previous study used Drop-seq to analyze the K562 cells treated with multiple drugs, including imatinib, rapamycin, and vinorelbine. When comparing the gene expression data from the two methods, we preferentially eliminated the batch effect in the data (Supplementary Fig. 4c). We observed a strong correlation between the two methods (coefficient of determination *R*^2^ > 0.9 for all three drug conditions; Fig. 3d, Supplementary Fig. 4d). When we compared the data among the different drug conditions, we found that the gene expression between rapamycin and vinorelbine had a stronger correlation than that between imatinib and the other drugs, and that pattern was the same in the MAPS-seq data and the Drop-seq data (Supplementary Fig. 4e). These results indicate that MAPS-seq produces data comparable to those of other scRNA-seq methods.

Consequently, we demonstrated that with our method, transcriptional changes in drug-treated cells can be analyzed at single-cell resolution, and marker genes that indicate the response of cells to each drug can be identified.

## Discussion

Investigation of transcriptomes at single-cell resolution provides precise insight into cellular functions and thereby furthers the understanding of larger units such as tissues and organs. In this study, the samples were pooled prior to the RT step, thus reducing the reagents and labor normally required for microwell-based approaches from 96 wells to a single tube. In addition, the use of BCOs and streptavidin-coated magnetic beads enabled most steps (such as RT, cDNA amplification, and tagmentation) to be processed with less labor. The beads were efficiently cleaned between each step, and the cost of MAPS-seq per cell was less than $1 (Supplementary Table 5).

Several other aspects of the MAPS-seq method make it an appealing choice for single-cell RNA sequencing. First, cDNA preparation by the PCR-based template-switching method is experimentally easier than that of the IVT method^31^. Second, MAPS-seq selectively sequences the 3′ end of the transcripts to obtain transcript data more cost-effectively than whole-transcriptome sequencing^32^. Finally, transposition with the Tn5 transposase in MAPS-seq makes that method more convenient than other methods because transposition proceeds in one step rather than by the two-step chemical fragmentation required in other methods^33,34^.

To validate the performance of MAPS-seq, we mixed two different cell lines and confirmed that little mRNA exchange between cells occurred when the cells were pooled. Based on the negative controls, we presume that the small amount of mRNA exchange was caused by uncaptured BCOs due to FACS error before the pooling step. We then demonstrated that MAPS-seq can be used to produce multiplexed cell drug response data by using the method in a drug response experiment. Notably, we retained data for 82% of the initial sorted cells after gene number filtering; such a high retention rate is difficult to achieve in a multiplexed drug screening experiment when conventional high-throughput methods are used. However, MAPS-seq can be used with high-quality cells because FACS selects and sorts live cells, excluding debris, from a population of drug-treated cells. Finally, we observed differentially expressed genes among the K562 cells treated with one of three drugs or DMSO. When comparing data from the imatinib-treated cells to the control data, we could observe the response of K562 to imatinib by analyzing the change in the expression level of related genes. We identified marker genes that exhibited transcriptional changes consistent with the mechanism of each drug, thus demonstrating that the MAPS-seq method is effective. Moreover, by revealing transcriptional changes among samples from the same cell line, these results showed the fine resolution of our method.

We believe that MAPS-seq is optimal for biological research on samples containing small numbers of cells. For example, very small numbers of circulating tumor cells (CTCs) are present in blood samples from patients with cancer. MAPS-seq should be able to isolate single CTCs from blood samples using FACS. For another example, taste buds with taste receptors are composed of 50–100 cells^35^. Single-cell studies of taste receptors are limited by the small numbers of cells in the taste bud. MAPS-seq will provide an opportunity to perform single-cell studies on tissues in which the numbers of cells of interest are limited, such as taste buds. Furthermore, MAPS-seq will likely be more widely applicable once other conditions are tested. For example, we expect that methanol-fixed cells and cell nuclei will be compatible with the method. Consequently, the potential of MAPS-seq is broader than the situations shown in this work. As an accessible, low-cost, and efficient method, MAPS-seq will be a useful addition to the techniques that have emerged in the era of single-cell study.

## Supplementary information

Supplementary data
