# Supplementary material for: MAPS-seq: magnetic bead-assisted parallel single-cell gene expression profiling
Source: Exp Mol Med. 2020 May 13;52(5):804–14. doi: 10.1038/s12276-020-0433-x (PMC7272464; doi:10.1038/s12276-020-0433-x)
Supplement: Supplementary file 1 — Supplementary data [file 12276_2020_433_MOESM1_ESM.docx]

**
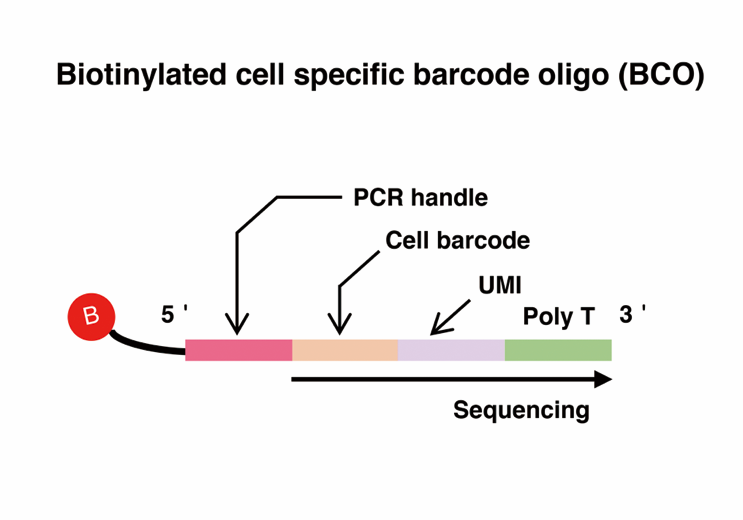
**

**Supplementary Figure 1.** Schematic of biotinylated cell specific barcode oligo. Sequencing starting point is the cell barcode and proceeds in the direction indicated by the arrow. B: Biotin molecule.


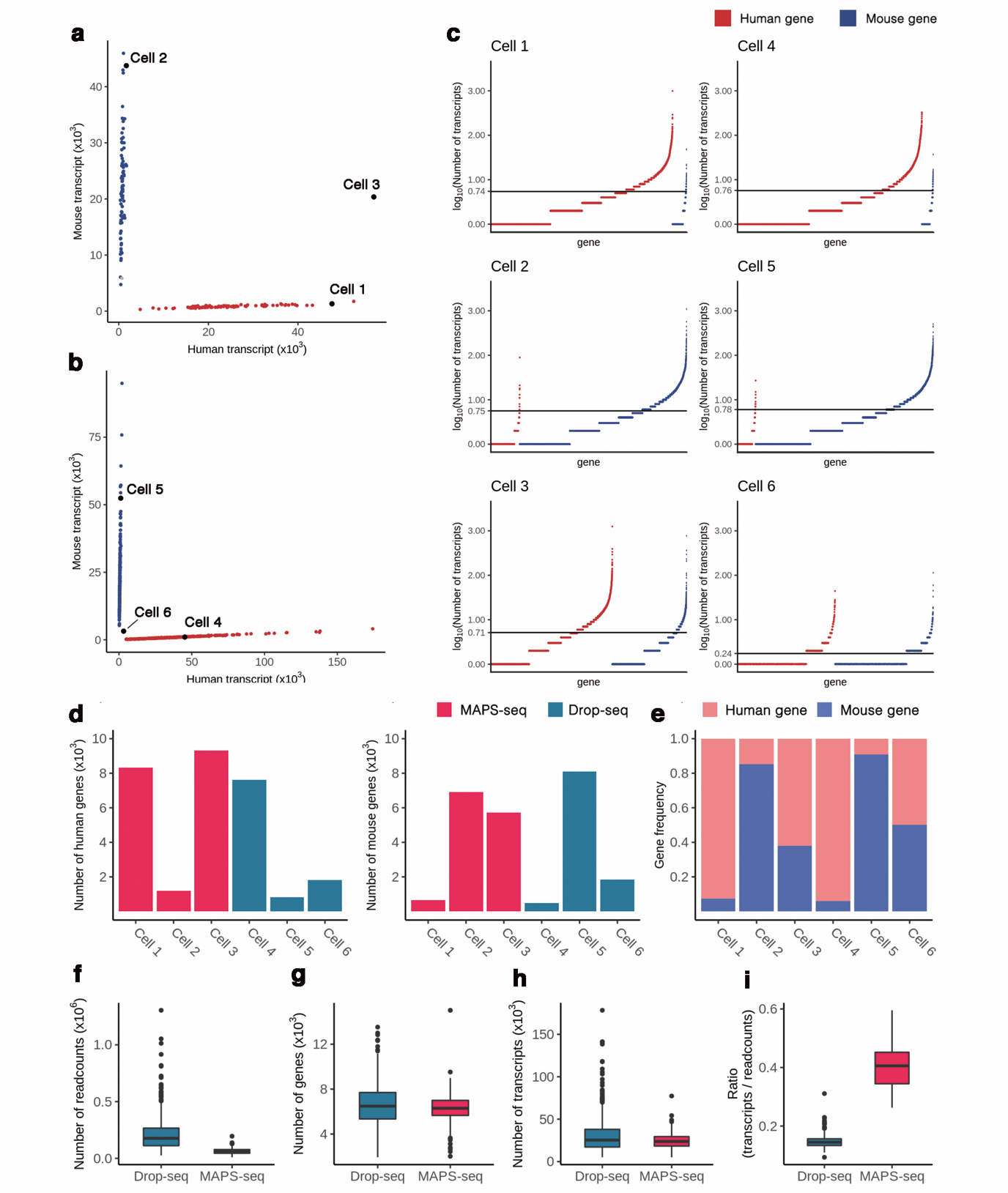


**Supplementary Figure 2.** Comparison of data trends between MAPS-seq and Drop-seq. (a) Numbers of human and mouse transcripts from 181 single cells in species-mixing MAPS-seq experiments. The red dots are identified as human cells, the blue dots are identified as mouse cells, and the gray dots are unidentified cells. The x-axis is the number of human transcripts, and the y-axis is the number of mouse transcripts. One human cell (Cell 1), one mouse cell (Cell 2), and one unidentified cell (Cell 3) were selected. (b) Numbers of human and mouse transcripts from 589 single cells in species-mixing Drop-seq experiments. The settings for the plot are the same as in (a). One human cell (Cell 4), one mouse cell (Cell 5), and one unidentified cell (Cell 6) were selected. (c) Dot plots of the gene-transcript numbers of each cell selected in (a, b). The x-axis is the gene name, and the y-axis is the log_10_(transcript number of each gene). Red dots represent human gene, and blue dots represent mouse genes. The gray horizontal line represents log_10_(mean number of transcripts) for all the genes in one cell. (d) Bar plots presenting the numbers of human (left) and mouse genes (right) in the cells selected in (a, b). (e) Bar plot presenting the frequencies of human and mouse genes in the cells selected in (a, b). The red bar represents the human genes, and the blue bar represents the mouse genes. (f) Boxplot showing the number of readcounts from MAPS-seq and Drop-seq. (g) Boxplot showing the number of genes identified from MAPS-seq and Drop-seq. (h) Boxplot showing the number of transcripts identified from MAPS-seq and Drop-seq. (i) Boxplot showing the ratio (number of transcripts / number of readcounts) for MAPS-seq and Drop-seq.

**
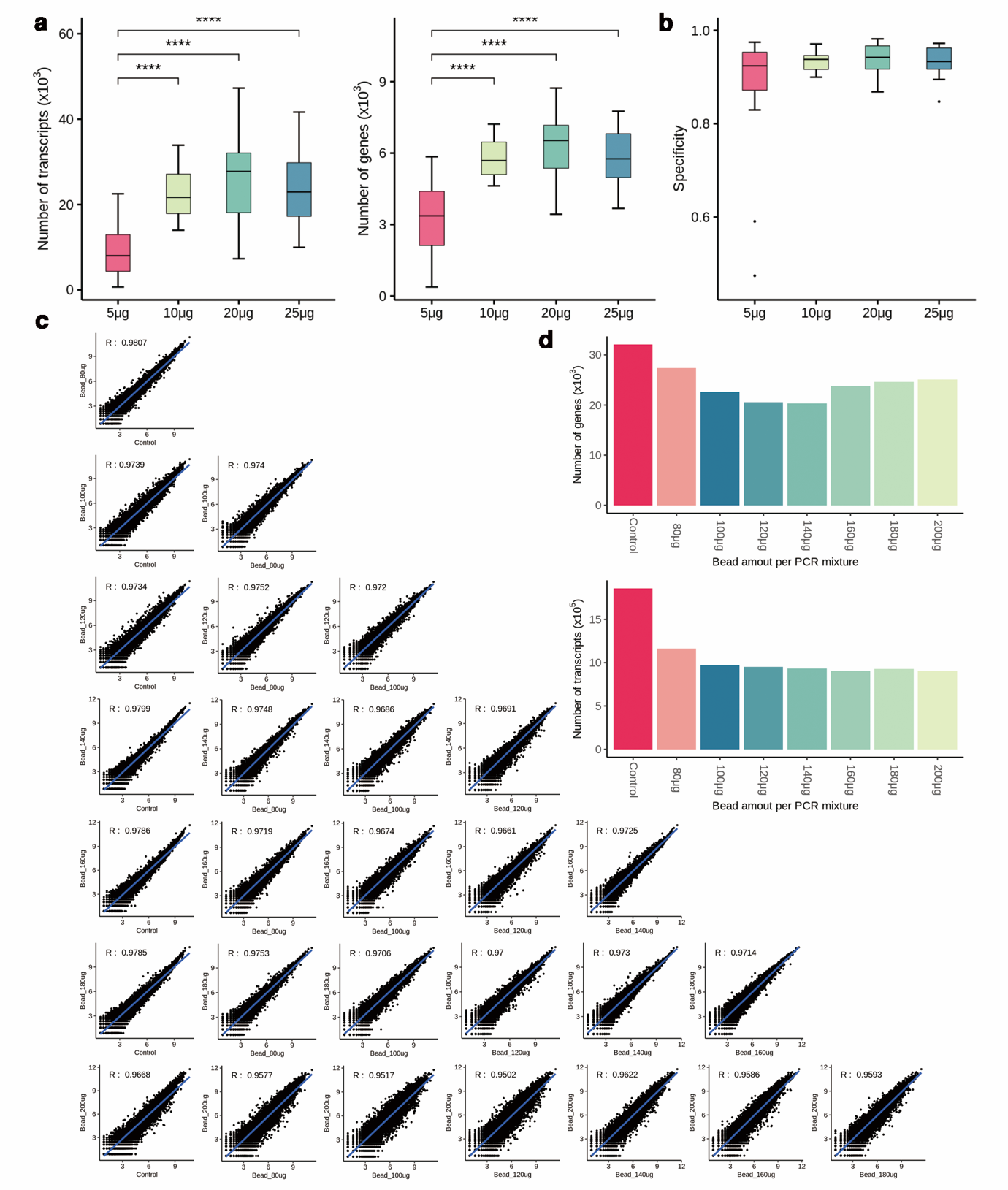
**

**Supplementary Figure 3.** Additional data for experiments to optimize the amount of streptavidin beads used in each well (5, 10, 20, or 25 μg beads per well). (a) Boxplots representing the number of transcripts (left) and genes (right) obtained for each condition. Significance (****) is indicated when p ≤ 0.0001. (b) Species specificity of each cell as shown by a simple species-mixing experiment. Eight cells each of HEK293T and NIH/3T3 were used for each condition. (c) Gene expression correlation for optimization experiments in the amount of streptavidin beads per reverse transcriptase mixture. Shown are Pearson correlations between converted gene expression values (log_2_ (CPM+1)) of eight conditions. (d) Bar plots representing the number of transcripts (top) and genes (bottom) obtained under each condition.

**
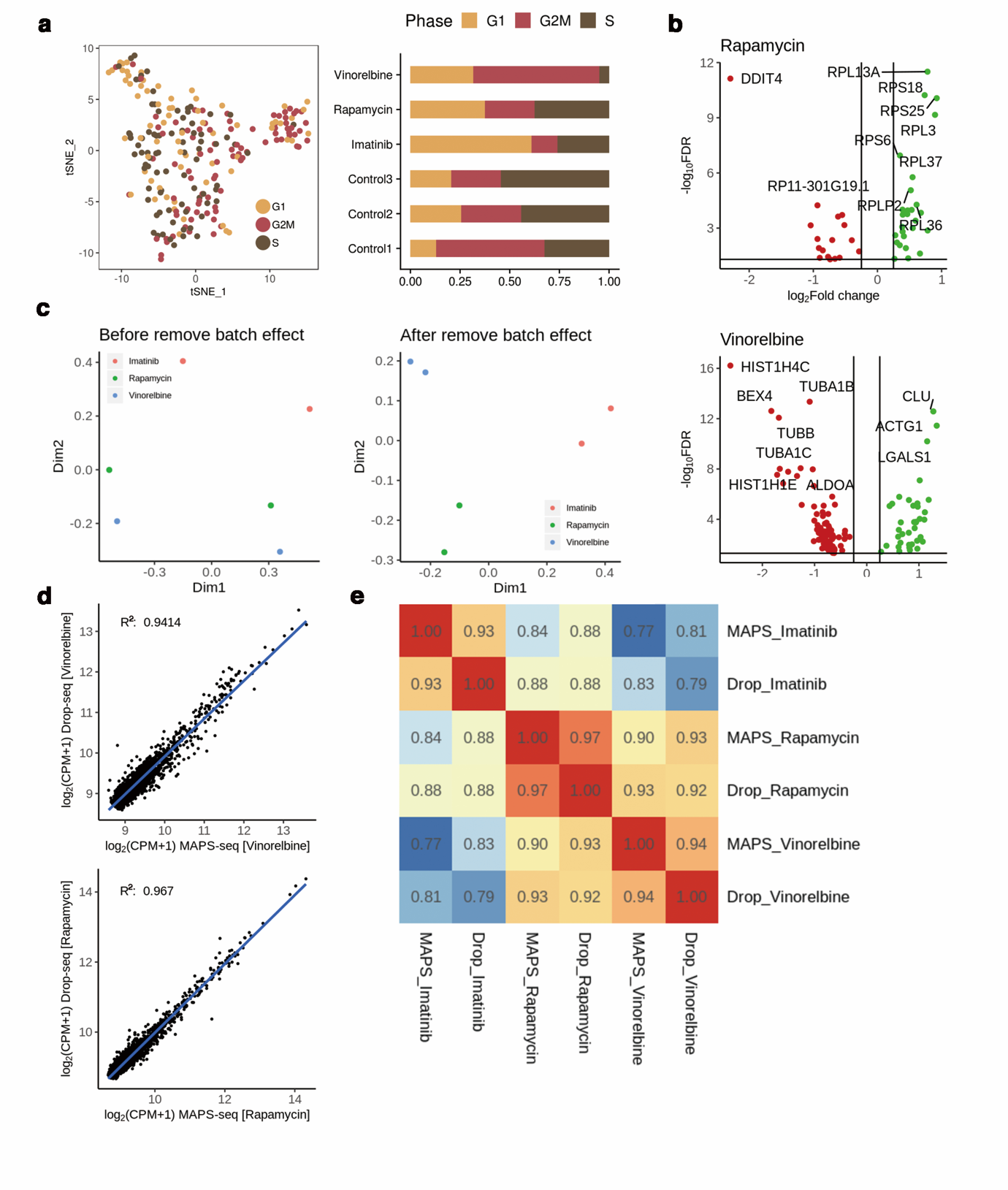
Supplementary Figure 4.** Additional data for drug treatment experiments. (a) Cell cycle state of each cell overlapped on t-SNE plot after cell cycle scoring (left); ratio of cell cycle states (G1 / G2M / S) in each sample (right). (b) Volcano plots displaying differentially expressed genes of rapamycin-treated (top) and vinorelbine-treated (bottom) cells compared with DMSO-treated controls. Genes with a p-value < 0.05 and an absolute value of log_2_ fold change (FC) > 0.25 are considered significant. Upregulated genes are green, down-regulated genes are red, and non-critical genes are gray. The 10 genes with the lowest p-values are labeled. (c) MDS plots before (left) and after (right) removal of the batch effect between data obtained by two methods. The batch effect was removed using the removeBatchEffect function of R package edgeR. Dimension 1 (Dim1) and dimension 2 (Dim2) are determined by Euclidean distance and represent typical log_2_ FC between samples. (d) Gene expression correlation between MAPS-seq and Drop-seq for rapamycin (top) and vinorelbine (bottom). Shown are coefficients of determination between two drugs under the same conditions in both methods. (e) Heatmap of gene expression correlations between different drugs and different methods. The number in the heatmap box is the coefficient of determination R^2^ between each condition.

**Supplementary Table 1.** Sequences of oligos used in this study

| **Oligo Name** | **Sequence 5′ to 3′** |
| --- | --- |
| Biotinylated cell specific barcode oligo (BCO) | 5**′**- /Biotin/AGTGGTATCAACGCAGAGTACJJJJJJNNNNNNN(T)_26_ -3**′** |
| Template-switching oligo (TSO) | 5**′** - AAGCAGTGGTATCAACGCAGAGTGAATrGrGrG - 3**′** |
| SMART PCR Primer | 5**′**- AAGCAGTGGTATCAACGCAGAGT - 3**′** |
| New P5-SMART PCR Hybrid Oligo | 5**′** - AATGATACGGCGACCACCGAGATCTACACGCCT GTCCGCGGAAGCAGTGGTATCAACGCAGAGT* A*C - 3**′** |
| Custom Read 1 Primer | 5**′** - GCCTGTCCGCGGAAGCAGTGGTATCAACGCAGAGTAC- 3**′** |

**Supplementary Table 2.** Biotinylated cell-specific barcode oligo (BCO) sequences. NNNNNN represents unique molecular identifier (UMI) sequences.

| **Oligo number** | **Cell barcode** | **Oligo Sequence** |
| --- | --- | --- |
| 1 | ACTCGT | /5Biotin/AGTGGTATCAACGCAGAGTACACTCGTNNNNNNNTTTTTTTTTTTTTTTTTTTTTTTTTT |
| 2 | TAGTCC | /5Biotin/AGTGGTATCAACGCAGAGTACTAGTCCNNNNNNNTTTTTTTTTTTTTTTTTTTTTTTTTT |
| 3 | CTATGC | /5Biotin/AGTGGTATCAACGCAGAGTACCTATGCNNNNNNNTTTTTTTTTTTTTTTTTTTTTTTTTT |
| 4 | CATACG | /5Biotin/AGTGGTATCAACGCAGAGTACCATACGNNNNNNNTTTTTTTTTTTTTTTTTTTTTTTTTT |
| 5 | TGACTC | /5Biotin/AGTGGTATCAACGCAGAGTACTGACTCNNNNNNNTTTTTTTTTTTTTTTTTTTTTTTTTT |
| 6 | ATTGCG | /5Biotin/AGTGGTATCAACGCAGAGTACATTGCGNNNNNNNTTTTTTTTTTTTTTTTTTTTTTTTTT |
| 7 | GCAATG | /5Biotin/AGTGGTATCAACGCAGAGTACGCAATGNNNNNNNTTTTTTTTTTTTTTTTTTTTTTTTTT |
| 8 | GTGACT | /5Biotin/AGTGGTATCAACGCAGAGTACGTGACTNNNNNNNTTTTTTTTTTTTTTTTTTTTTTTTTT |
| 9 | AGTCTG | /5Biotin/AGTGGTATCAACGCAGAGTACAGTCTGNNNNNNNTTTTTTTTTTTTTTTTTTTTTTTTTT |
| 10 | GCCTTA | /5Biotin/AGTGGTATCAACGCAGAGTACGCCTTANNNNNNNTTTTTTTTTTTTTTTTTTTTTTTTTT |
| 11 | CCTGAT | /5Biotin/AGTGGTATCAACGCAGAGTACCCTGATNNNNNNNTTTTTTTTTTTTTTTTTTTTTTTTTT |
| 12 | GGCATT | /5Biotin/AGTGGTATCAACGCAGAGTACGGCATTNNNNNNNTTTTTTTTTTTTTTTTTTTTTTTTTT |
| 13 | AGATCC | /5Biotin/AGTGGTATCAACGCAGAGTACAGATCCNNNNNNNTTTTTTTTTTTTTTTTTTTTTTTTTT |
| 14 | TCACAG | /5Biotin/AGTGGTATCAACGCAGAGTACTCACAGNNNNNNNTTTTTTTTTTTTTTTTTTTTTTTTTT |
| 15 | TCAGCA | /5Biotin/AGTGGTATCAACGCAGAGTACTCAGCANNNNNNNTTTTTTTTTTTTTTTTTTTTTTTTTT |
| 16 | CTCAAG | /5Biotin/AGTGGTATCAACGCAGAGTACCTCAAGNNNNNNNTTTTTTTTTTTTTTTTTTTTTTTTTT |
| 17 | CCGATA | /5Biotin/AGTGGTATCAACGCAGAGTACCCGATANNNNNNNTTTTTTTTTTTTTTTTTTTTTTTTTT |
| 18 | CAAGGT | /5Biotin/AGTGGTATCAACGCAGAGTACCAAGGTNNNNNNNTTTTTTTTTTTTTTTTTTTTTTTTTT |
| 19 | GTAGTC | /5Biotin/AGTGGTATCAACGCAGAGTACGTAGTCNNNNNNNTTTTTTTTTTTTTTTTTTTTTTTTTT |
| 20 | TACCGT | /5Biotin/AGTGGTATCAACGCAGAGTACTACCGTNNNNNNNTTTTTTTTTTTTTTTTTTTTTTTTTT |
| 21 | GGTTCA | /5Biotin/AGTGGTATCAACGCAGAGTACGGTTCANNNNNNNTTTTTTTTTTTTTTTTTTTTTTTTTT |
| 22 | ACGGTT | /5Biotin/AGTGGTATCAACGCAGAGTACACGGTTNNNNNNNTTTTTTTTTTTTTTTTTTTTTTTTTT |
| 23 | GACGAT | /5Biotin/AGTGGTATCAACGCAGAGTACGACGATNNNNNNNTTTTTTTTTTTTTTTTTTTTTTTTTT |
| 24 | GCTAGA | /5Biotin/AGTGGTATCAACGCAGAGTACGCTAGANNNNNNNTTTTTTTTTTTTTTTTTTTTTTTTTT |
| 25 | GATCTC | /5Biotin/AGTGGTATCAACGCAGAGTACGATCTCNNNNNNNTTTTTTTTTTTTTTTTTTTTTTTTTT |
| 26 | ATCAGC | /5Biotin/AGTGGTATCAACGCAGAGTACATCAGCNNNNNNNTTTTTTTTTTTTTTTTTTTTTTTTTT |
| 27 | CTGTCA | /5Biotin/AGTGGTATCAACGCAGAGTACCTGTCANNNNNNNTTTTTTTTTTTTTTTTTTTTTTTTTT |
| 28 | GCTTAG | /5Biotin/AGTGGTATCAACGCAGAGTACGCTTAGNNNNNNNTTTTTTTTTTTTTTTTTTTTTTTTTT |
| 29 | AAGCCT | /5Biotin/AGTGGTATCAACGCAGAGTACAAGCCTNNNNNNNTTTTTTTTTTTTTTTTTTTTTTTTTT |
| 30 | TTCGGA | /5Biotin/AGTGGTATCAACGCAGAGTACTTCGGANNNNNNNTTTTTTTTTTTTTTTTTTTTTTTTTT |
| 31 | AACTCG | /5Biotin/AGTGGTATCAACGCAGAGTACAACTCGNNNNNNNTTTTTTTTTTTTTTTTTTTTTTTTTT |
| 32 | GTGTAC | /5Biotin/AGTGGTATCAACGCAGAGTACGTGTACNNNNNNNTTTTTTTTTTTTTTTTTTTTTTTTTT |
| 33 | ACATGG | /5Biotin/AGTGGTATCAACGCAGAGTACACATGGNNNNNNNTTTTTTTTTTTTTTTTTTTTTTTTTT |
| 34 | CGTGTA | /5Biotin/AGTGGTATCAACGCAGAGTACCGTGTANNNNNNNTTTTTTTTTTTTTTTTTTTTTTTTTT |
| 35 | TAGCTG | /5Biotin/AGTGGTATCAACGCAGAGTACTAGCTGNNNNNNNTTTTTTTTTTTTTTTTTTTTTTTTTT |
| 36 | CGATTG | /5Biotin/AGTGGTATCAACGCAGAGTACCGATTGNNNNNNNTTTTTTTTTTTTTTTTTTTTTTTTTT |
| 37 | GGACAT | /5Biotin/AGTGGTATCAACGCAGAGTACGGACATNNNNNNNTTTTTTTTTTTTTTTTTTTTTTTTTT |
| 38 | ATGCAG | /5Biotin/AGTGGTATCAACGCAGAGTACATGCAGNNNNNNNTTTTTTTTTTTTTTTTTTTTTTTTTT |
| 39 | AGCTGT | /5Biotin/AGTGGTATCAACGCAGAGTACAGCTGTNNNNNNNTTTTTTTTTTTTTTTTTTTTTTTTTT |
| 40 | AATGGC | /5Biotin/AGTGGTATCAACGCAGAGTACAATGGCNNNNNNNTTTTTTTTTTTTTTTTTTTTTTTTTT |
| 41 | TCGTGA | /5Biotin/AGTGGTATCAACGCAGAGTACTCGTGANNNNNNNTTTTTTTTTTTTTTTTTTTTTTTTTT |
| 42 | GTGCTA | /5Biotin/AGTGGTATCAACGCAGAGTACGTGCTANNNNNNNTTTTTTTTTTTTTTTTTTTTTTTTTT |
| 43 | GGTAAC | /5Biotin/AGTGGTATCAACGCAGAGTACGGTAACNNNNNNNTTTTTTTTTTTTTTTTTTTTTTTTTT |
| 44 | CAGTAG | /5Biotin/AGTGGTATCAACGCAGAGTACCAGTAGNNNNNNNTTTTTTTTTTTTTTTTTTTTTTTTTT |
| 45 | CGAACT | /5Biotin/AGTGGTATCAACGCAGAGTACCGAACTNNNNNNNTTTTTTTTTTTTTTTTTTTTTTTTTT |
| 46 | CACTGA | /5Biotin/AGTGGTATCAACGCAGAGTACCACTGANNNNNNNTTTTTTTTTTTTTTTTTTTTTTTTTT |
| 47 | CTTCGA | /5Biotin/AGTGGTATCAACGCAGAGTACCTTCGANNNNNNNTTTTTTTTTTTTTTTTTTTTTTTTTT |
| 48 | TGCACA | /5Biotin/AGTGGTATCAACGCAGAGTACTGCACANNNNNNNTTTTTTTTTTTTTTTTTTTTTTTTTT |
| 49 | TCGAAC | /5Biotin/AGTGGTATCAACGCAGAGTACTCGAACNNNNNNNTTTTTTTTTTTTTTTTTTTTTTTTTT |
| 50 | TGGCAA | /5Biotin/AGTGGTATCAACGCAGAGTACTGGCAANNNNNNNTTTTTTTTTTTTTTTTTTTTTTTTTT |
| 51 | GTATCG | /5Biotin/AGTGGTATCAACGCAGAGTACGTATCGNNNNNNNTTTTTTTTTTTTTTTTTTTTTTTTTT |
| 52 | AGGATC | /5Biotin/AGTGGTATCAACGCAGAGTACAGGATCNNNNNNNTTTTTTTTTTTTTTTTTTTTTTTTTT |
| 53 | TGCTAG | /5Biotin/AGTGGTATCAACGCAGAGTACTGCTAGNNNNNNNTTTTTTTTTTTTTTTTTTTTTTTTTT |
| 54 | TACGTC | /5Biotin/AGTGGTATCAACGCAGAGTACTACGTCNNNNNNNTTTTTTTTTTTTTTTTTTTTTTTTTT |
| 55 | ACAGAC | /5Biotin/AGTGGTATCAACGCAGAGTACACAGACNNNNNNNTTTTTTTTTTTTTTTTTTTTTTTTTT |
| 56 | TACAGC | /5Biotin/AGTGGTATCAACGCAGAGTACTACAGCNNNNNNNTTTTTTTTTTTTTTTTTTTTTTTTTT |
| 57 | ACTGAG | /5Biotin/AGTGGTATCAACGCAGAGTACACTGAGNNNNNNNTTTTTTTTTTTTTTTTTTTTTTTTTT |
| 58 | CACACT | /5Biotin/AGTGGTATCAACGCAGAGTACCACACTNNNNNNNTTTTTTTTTTTTTTTTTTTTTTTTTT |
| 59 | GTGAGA | /5Biotin/AGTGGTATCAACGCAGAGTACGTGAGANNNNNNNTTTTTTTTTTTTTTTTTTTTTTTTTT |
| 60 | AGCTCA | /5Biotin/AGTGGTATCAACGCAGAGTACAGCTCANNNNNNNTTTTTTTTTTTTTTTTTTTTTTTTTT |
| 61 | TGCATG | /5Biotin/AGTGGTATCAACGCAGAGTACTGCATGNNNNNNNTTTTTTTTTTTTTTTTTTTTTTTTTT |
| 62 | ACGACT | /5Biotin/AGTGGTATCAACGCAGAGTACACGACTNNNNNNNTTTTTTTTTTTTTTTTTTTTTTTTTT |
| 63 | AGACAC | /5Biotin/AGTGGTATCAACGCAGAGTACAGACACNNNNNNNTTTTTTTTTTTTTTTTTTTTTTTTTT |
| 64 | TCTGTC | /5Biotin/AGTGGTATCAACGCAGAGTACTCTGTCNNNNNNNTTTTTTTTTTTTTTTTTTTTTTTTTT |
| 65 | AGTGTC | /5Biotin/AGTGGTATCAACGCAGAGTACAGTGTCNNNNNNNTTTTTTTTTTTTTTTTTTTTTTTTTT |
| 66 | GATGAG | /5Biotin/AGTGGTATCAACGCAGAGTACGATGAGNNNNNNNTTTTTTTTTTTTTTTTTTTTTTTTTT |
| 67 | GATAGC | /5Biotin/AGTGGTATCAACGCAGAGTACGATAGCNNNNNNNTTTTTTTTTTTTTTTTTTTTTTTTTT |
| 68 | TCACGA | /5Biotin/AGTGGTATCAACGCAGAGTACTCACGANNNNNNNTTTTTTTTTTTTTTTTTTTTTTTTTT |
| 69 | GCATCA | /5Biotin/AGTGGTATCAACGCAGAGTACGCATCANNNNNNNTTTTTTTTTTTTTTTTTTTTTTTTTT |
| 70 | CGCTAT | /5Biotin/AGTGGTATCAACGCAGAGTACCGCTATNNNNNNNTTTTTTTTTTTTTTTTTTTTTTTTTT |
| 71 | CATCAG | /5Biotin/AGTGGTATCAACGCAGAGTACCATCAGNNNNNNNTTTTTTTTTTTTTTTTTTTTTTTTTT |
| 72 | CACGTA | /5Biotin/AGTGGTATCAACGCAGAGTACCACGTANNNNNNNTTTTTTTTTTTTTTTTTTTTTTTTTT |
| 73 | GACTCA | /5Biotin/AGTGGTATCAACGCAGAGTACGACTCANNNNNNNTTTTTTTTTTTTTTTTTTTTTTTTTT |
| 74 | TCATGC | /5Biotin/AGTGGTATCAACGCAGAGTACTCATGCNNNNNNNTTTTTTTTTTTTTTTTTTTTTTTTTT |
| 75 | CAGAGA | /5Biotin/AGTGGTATCAACGCAGAGTACCAGAGANNNNNNNTTTTTTTTTTTTTTTTTTTTTTTTTT |
| 76 | GAGCAT | /5Biotin/AGTGGTATCAACGCAGAGTACGAGCATNNNNNNNTTTTTTTTTTTTTTTTTTTTTTTTTT |
| 77 | CTGATC | /5Biotin/AGTGGTATCAACGCAGAGTACCTGATCNNNNNNNTTTTTTTTTTTTTTTTTTTTTTTTTT |
| 78 | GCATGT | /5Biotin/AGTGGTATCAACGCAGAGTACGCATGTNNNNNNNTTTTTTTTTTTTTTTTTTTTTTTTTT |
| 79 | GAGTGT | /5Biotin/AGTGGTATCAACGCAGAGTACGAGTGTNNNNNNNTTTTTTTTTTTTTTTTTTTTTTTTTT |
| 80 | TGAGCT | /5Biotin/AGTGGTATCAACGCAGAGTACTGAGCTNNNNNNNTTTTTTTTTTTTTTTTTTTTTTTTTT |
| 81 | GAGATG | /5Biotin/AGTGGTATCAACGCAGAGTACGAGATGNNNNNNNTTTTTTTTTTTTTTTTTTTTTTTTTT |
| 82 | ACGCTA | /5Biotin/AGTGGTATCAACGCAGAGTACACGCTANNNNNNNTTTTTTTTTTTTTTTTTTTTTTTTTT |
| 83 | TGACGT | /5Biotin/AGTGGTATCAACGCAGAGTACTGACGTNNNNNNNTTTTTTTTTTTTTTTTTTTTTTTTTT |
| 84 | AGCACT | /5Biotin/AGTGGTATCAACGCAGAGTACAGCACTNNNNNNNTTTTTTTTTTTTTTTTTTTTTTTTTT |
| 85 | GCTATC | /5Biotin/AGTGGTATCAACGCAGAGTACGCTATCNNNNNNNTTTTTTTTTTTTTTTTTTTTTTTTTT |
| 86 | ATGACG | /5Biotin/AGTGGTATCAACGCAGAGTACATGACGNNNNNNNTTTTTTTTTTTTTTTTTTTTTTTTTT |
| 87 | CTACGT | /5Biotin/AGTGGTATCAACGCAGAGTACCTACGTNNNNNNNTTTTTTTTTTTTTTTTTTTTTTTTTT |
| 88 | CACATC | /5Biotin/AGTGGTATCAACGCAGAGTACCACATCNNNNNNNTTTTTTTTTTTTTTTTTTTTTTTTTT |
| 89 | TGTGTG | /5Biotin/AGTGGTATCAACGCAGAGTACTGTGTGNNNNNNNTTTTTTTTTTTTTTTTTTTTTTTTTT |
| 90 | TGTCGA | /5Biotin/AGTGGTATCAACGCAGAGTACTGTCGANNNNNNNTTTTTTTTTTTTTTTTTTTTTTTTTT |
| 91 | ACGATG | /5Biotin/AGTGGTATCAACGCAGAGTACACGATGNNNNNNNTTTTTTTTTTTTTTTTTTTTTTTTTT |
| 92 | TACGAG | /5Biotin/AGTGGTATCAACGCAGAGTACTACGAGNNNNNNNTTTTTTTTTTTTTTTTTTTTTTTTTT |
| 93 | CGTAGA | /5Biotin/AGTGGTATCAACGCAGAGTACCGTAGANNNNNNNTTTTTTTTTTTTTTTTTTTTTTTTTT |
| 94 | ATGCTC | /5Biotin/AGTGGTATCAACGCAGAGTACATGCTCNNNNNNNTTTTTTTTTTTTTTTTTTTTTTTTTT |
| 95 | ACACTC | /5Biotin/AGTGGTATCAACGCAGAGTACACACTCNNNNNNNTTTTTTTTTTTTTTTTTTTTTTTTTT |
| 96 | TGAGAG | /5Biotin/AGTGGTATCAACGCAGAGTACTGAGAGNNNNNNNTTTTTTTTTTTTTTTTTTTTTTTTTT |

**Supplementary Table 3.** Differential gene list for each drug.

p_value: p-value of the gene between the two groups using the Wilcoxon Rank Sum test; avg_logFC: log fold change of the average expression between the two groups, positive values indicate that the gene is more highly expressed in the first group; pct.1: the percentage of cells where the gene is detected in the drug groups; pct.2: the percentage of cells where the gene is detected in the control groups; p_val_adj: adjusted p-value, based on Bonferroni correction using all genes in the dataset.

| **p_value** | **avg_logFC** | **pct.1** | **pct.2** | **p_val_adj** | **gene** | **ident** |
| --- | --- | --- | --- | --- | --- | --- |
| 2.69E-17 | 2.46399593 | 1 | 0.286 | 4.20E-13 | GYPB | Imatinib |
| 3.40E-14 | 2.6742314 | 1 | 0.699 | 5.30E-10 | HBZ | Imatinib |
| 6.45E-14 | 2.35638144 | 1 | 0.932 | 1.01E-09 | HBA1 | Imatinib |
| 1.38E-13 | 1.68673597 | 1 | 1 | 2.16E-09 | HBG2 | Imatinib |
| 6.82E-13 | 1.48891569 | 1 | 1 | 1.06E-08 | HBG1 | Imatinib |
| 6.39E-12 | -1.4550846 | 0.478 | 0.985 | 9.97E-08 | ENO1 | Imatinib |
| 8.91E-12 | -1.0183132 | 0.913 | 1 | 1.39E-07 | SLC25A5 | Imatinib |
| 1.58E-11 | 2.42724991 | 1 | 0.895 | 2.46E-07 | HBA2 | Imatinib |
| 1.25E-10 | -1.0497211 | 0.783 | 0.985 | 1.94E-06 | GAPDH | Imatinib |
| 2.55E-10 | -1.3043771 | 0.435 | 0.955 | 3.98E-06 | HSPD1 | Imatinib |
| 3.07E-10 | -1.7267228 | 0.13 | 0.872 | 4.78E-06 | LDHA | Imatinib |
| 4.42E-10 | -0.8701357 | 0.957 | 1 | 6.90E-06 | ATP5G3 | Imatinib |
| 9.46E-10 | -0.8573287 | 0.957 | 1 | 1.48E-05 | HSP90AB1 | Imatinib |
| 2.10E-09 | -1.5242963 | 0.13 | 0.925 | 3.28E-05 | NMU | Imatinib |
| 2.17E-09 | -1.2959547 | 0.391 | 0.91 | 3.38E-05 | RANBP1 | Imatinib |
| 4.89E-09 | -0.9635562 | 0.826 | 0.992 | 7.62E-05 | HSP90AA1 | Imatinib |
| 8.72E-09 | -1.8134206 | 0.13 | 0.827 | 0.00013598 | DDIT4 | Imatinib |
| 1.12E-08 | -1.1683854 | 0.435 | 0.947 | 0.00017424 | TPI1 | Imatinib |
| 1.26E-08 | -1.2055819 | 0.391 | 0.887 | 0.00019618 | CACYBP | Imatinib |
| 1.42E-08 | 1.35304892 | 0.957 | 0.774 | 0.00022183 | ATF7IP2 | Imatinib |
| 1.67E-08 | -1.2931035 | 0.043 | 0.737 | 0.00026099 | SCD | Imatinib |
| 1.72E-08 | -1.3864483 | 0.087 | 0.835 | 0.0002687 | DDX21 | Imatinib |
| 1.96E-08 | -1.2584288 | 0.304 | 0.887 | 0.0003057 | CCT6A | Imatinib |
| 2.01E-08 | 0.98415979 | 0.957 | 0.962 | 0.0003136 | BLVRB | Imatinib |
| 2.28E-08 | -1.1326205 | 0.304 | 0.91 | 0.00035496 | NME1 | Imatinib |
| 2.50E-08 | -1.3577026 | 0.087 | 0.782 | 0.00038951 | PKM | Imatinib |
| 2.53E-08 | -1.083563 | 0.435 | 0.917 | 0.00039486 | PTMA | Imatinib |
| 2.72E-08 | -0.8266702 | 0.826 | 1 | 0.00042443 | TUBA1B | Imatinib |
| 2.74E-08 | -1.0449287 | 0.435 | 0.97 | 0.00042799 | NPM1 | Imatinib |
| 3.47E-08 | -1.0970966 | 0.435 | 0.985 | 0.00054122 | ALDOA | Imatinib |
| 3.80E-08 | -1.0976457 | 0.391 | 0.917 | 0.00059312 | ATP5B | Imatinib |
| 4.45E-08 | -1.3201762 | 0.043 | 0.767 | 0.00069473 | PAICS | Imatinib |
| 4.52E-08 | -1.3195348 | 0.13 | 0.805 | 0.0007058 | FBL | Imatinib |
| 4.59E-08 | 1.66170844 | 0.826 | 0.556 | 0.00071563 | SLC25A37 | Imatinib |
| 5.49E-08 | 0.80303289 | 0.261 | 0.008 | 0.00085605 | AHSP | Imatinib |
| 6.25E-08 | -0.8401004 | 0.696 | 0.992 | 0.00097565 | H2AFZ | Imatinib |
| 6.51E-08 | -1.0448694 | 0.304 | 0.91 | 0.00101581 | TAF9 | Imatinib |
| 6.52E-08 | 1.18043264 | 0.348 | 0.03 | 0.00101708 | MIR144 | Imatinib |
| 9.10E-08 | -1.145282 | 0.261 | 0.88 | 0.0014193 | NOL7 | Imatinib |
| 1.05E-07 | 0.76835798 | 1 | 1 | 0.00163178 | MT-RNR2 | Imatinib |
| 1.06E-07 | -1.1838481 | 0.13 | 0.782 | 0.00164964 | SNRPG | Imatinib |
| 1.06E-07 | -1.3116434 | 0.13 | 0.782 | 0.00164964 | ANP32E | Imatinib |
| 1.22E-07 | 1.91193809 | 0.652 | 0.241 | 0.00191066 | ALAS2 | Imatinib |
| 1.27E-07 | 0.30775729 | 1 | 1 | 0.00197656 | RPLP1 | Imatinib |
| 1.28E-07 | -1.0727636 | 0.043 | 0.729 | 0.00200373 | COA4 | Imatinib |
| 1.64E-07 | -1.2138927 | 0.087 | 0.752 | 0.00256271 | LYAR | Imatinib |
| 1.68E-07 | -1.1385467 | 0.304 | 0.88 | 0.00262757 | TFAM | Imatinib |
| 1.74E-07 | -0.9667333 | 0.13 | 0.835 | 0.00271993 | APRT | Imatinib |
| 1.76E-07 | -1.1290032 | 0.348 | 0.857 | 0.00274393 | SERBP1 | Imatinib |
| 1.78E-07 | -0.7010118 | 0.652 | 0.94 | 0.00277052 | LDHB | Imatinib |
| 2.12E-07 | -1.0927675 | 0.174 | 0.82 | 0.00331279 | SNHG16 | Imatinib |
| 2.39E-07 | -0.9800852 | 0.174 | 0.805 | 0.00373186 | MRPL41 | Imatinib |
| 2.94E-07 | -1.0980857 | 0.217 | 0.835 | 0.00459303 | MDH2 | Imatinib |
| 3.00E-07 | 0.51593244 | 1 | 0.992 | 0.00467619 | RPS19 | Imatinib |
| 3.36E-07 | -0.8978368 | 0.391 | 0.932 | 0.00524923 | TUBB | Imatinib |
| 3.70E-07 | -1.0341807 | 0.043 | 0.692 | 0.00576855 | SNRPA1 | Imatinib |
| 4.16E-07 | -1.3174989 | 0.043 | 0.684 | 0.00648913 | HMGN5 | Imatinib |
| 4.35E-07 | -0.9924802 | 0.261 | 0.842 | 0.00679355 | HSPA9 | Imatinib |
| 5.00E-07 | -0.9120177 | 0.087 | 0.752 | 0.00779381 | GTF3A | Imatinib |
| 5.49E-07 | -1.0217976 | 0.174 | 0.842 | 0.00855703 | MRPL22 | Imatinib |
| 5.58E-07 | -0.936575 | 0.304 | 0.88 | 0.00870262 | SNRPB | Imatinib |
| 6.37E-07 | -1.0082251 | 0.565 | 0.917 | 0.00993446 | NCL | Imatinib |
| 6.71E-07 | -1.1717737 | 0.043 | 0.662 | 0.01046786 | HSPA5 | Imatinib |
| 6.87E-07 | -0.9751047 | 0.043 | 0.692 | 0.01072068 | GNL3 | Imatinib |
| 6.91E-07 | -0.6772932 | 0.826 | 0.985 | 0.01078507 | RPLP0 | Imatinib |
| 7.38E-07 | -1.2059045 | 0.043 | 0.677 | 0.01151236 | MTHFD2 | Imatinib |
| 7.61E-07 | -1.0457244 | 0.304 | 0.88 | 0.01187732 | EEF1E1 | Imatinib |
| 7.92E-07 | -1.0458102 | 0.217 | 0.789 | 0.01235432 | PDIA6 | Imatinib |
| 8.07E-07 | -1.1558061 | 0.304 | 0.812 | 0.01259197 | UCHL5 | Imatinib |
| 8.08E-07 | -1.0782049 | 0.174 | 0.752 | 0.01260876 | TXNDC17 | Imatinib |
| 8.21E-07 | -1.0442013 | 0.217 | 0.835 | 0.01280347 | SRSF7 | Imatinib |
| 8.70E-07 | -1.1530438 | 0.087 | 0.677 | 0.01356781 | DDX39A | Imatinib |
| 8.74E-07 | -1.0470836 | 0.174 | 0.805 | 0.01363786 | GLUL | Imatinib |
| 9.34E-07 | -0.862935 | 0.609 | 0.97 | 0.01456962 | HSPE1 | Imatinib |
| 9.90E-07 | 0.44198431 | 1 | 1 | 0.01544057 | RPS14 | Imatinib |
| 1.00E-06 | -0.8267568 | 0.522 | 0.932 | 0.01562644 | PARK7 | Imatinib |
| 1.02E-06 | -0.9828276 | 0.217 | 0.842 | 0.01597371 | FDPS | Imatinib |
| 1.26E-06 | -0.8543978 | 0.522 | 0.932 | 0.01962545 | PRDX1 | Imatinib |
| 1.28E-06 | -1.056419 | 0.087 | 0.729 | 0.01992318 | TUFM | Imatinib |
| 1.31E-06 | -0.9976812 | 0.261 | 0.82 | 0.02048274 | TIMM17A | Imatinib |
| 1.39E-06 | -0.9763142 | 0.261 | 0.88 | 0.02166951 | SLIRP | Imatinib |
| 1.52E-06 | 1.03402826 | 1 | 1 | 0.02370078 | RNA28S5 | Imatinib |
| 1.55E-06 | -1.0397585 | 0.391 | 0.91 | 0.02416465 | TMSB4X | Imatinib |
| 1.58E-06 | -0.9155289 | 0.348 | 0.865 | 0.02457321 | EIF3M | Imatinib |
| 1.63E-06 | -0.9291215 | 0.087 | 0.714 | 0.02535214 | PPA2 | Imatinib |
| 1.67E-06 | -1.0362357 | 0.087 | 0.669 | 0.02599067 | EXOSC3 | Imatinib |
| 1.68E-06 | -0.9743535 | 0.13 | 0.774 | 0.02625643 | ITM2A | Imatinib |
| 1.72E-06 | -1.0799537 | 0.217 | 0.774 | 0.02682388 | NQO1 | Imatinib |
| 1.73E-06 | -0.9530181 | 0.174 | 0.827 | 0.02693028 | MRPL42 | Imatinib |
| 1.86E-06 | -0.9655297 | 0.174 | 0.722 | 0.02905858 | VDAC1 | Imatinib |
| 1.90E-06 | -0.9755484 | 0.174 | 0.789 | 0.02956607 | NPM3 | Imatinib |
| 2.08E-06 | -1.0014492 | 0.174 | 0.782 | 0.03239438 | NHP2 | Imatinib |
| 2.17E-06 | -0.9404493 | 0.174 | 0.797 | 0.03378439 | EIF3J | Imatinib |
| 2.19E-06 | -1.0324701 | 0.043 | 0.662 | 0.03414633 | PSMG1 | Imatinib |
| 2.26E-06 | -0.8683274 | 0.609 | 0.947 | 0.03524123 | POMP | Imatinib |
| 2.33E-06 | -1.0156908 | 0.13 | 0.752 | 0.0363924 | NDUFAF4 | Imatinib |
| 2.37E-06 | -0.9877122 | 0.174 | 0.752 | 0.03689619 | STOML2 | Imatinib |
| 2.40E-06 | -1.0779073 | 0.043 | 0.632 | 0.03741796 | SDF2L1 | Imatinib |
| 2.46E-06 | -0.9102999 | 0.478 | 0.917 | 0.03840693 | CYCS | Imatinib |
| 2.62E-06 | -0.9474407 | 0.478 | 0.872 | 0.04082019 | EIF3I | Imatinib |
| 2.68E-06 | -0.9683195 | 0 | 0.571 | 0.04173982 | TSEN15 | Imatinib |
| 2.68E-06 | -1.0767335 | 0 | 0.571 | 0.04173982 | EGR1 | Imatinib |
| 2.68E-06 | 0.42288916 | 1 | 1 | 0.04185227 | RPL31 | Imatinib |
| 2.70E-06 | -1.0676963 | 0.043 | 0.602 | 0.04217826 | TMEM147 | Imatinib |
| 2.87E-06 | -0.9159996 | 0.261 | 0.857 | 0.04474045 | NOP58 | Imatinib |
| 2.93E-06 | -0.6938549 | 0.087 | 0.684 | 0.04578566 | PRMT1 | Imatinib |
| 2.93E-06 | -0.7507121 | 0.087 | 0.684 | 0.04578566 | TIMM13 | Imatinib |
| 3.07E-06 | -0.8253034 | 0.043 | 0.654 | 0.04787583 | BZW2 | Imatinib |
| 3.16E-06 | -0.8066814 | 0.174 | 0.805 | 0.04933357 | SPCS1 | Imatinib |
| 3.20E-06 | -0.7961098 | 0.13 | 0.729 | 0.04987597 | NDUFA5 | Imatinib |
| 2.01E-16 | 0.78068357 | 1 | 0.977 | 3.13E-12 | RPL13A | Rapamycin |
| 4.75E-16 | -2.2937042 | 0 | 0.827 | 7.41E-12 | DDIT4 | Rapamycin |
| 3.79E-15 | 0.73649065 | 1 | 0.992 | 5.91E-11 | RPS18 | Rapamycin |
| 5.51E-15 | 0.92450035 | 1 | 0.857 | 8.60E-11 | RPS25 | Rapamycin |
| 4.45E-14 | 0.89799487 | 1 | 0.812 | 6.94E-10 | RPL3 | Rapamycin |
| 7.21E-12 | 0.35149969 | 1 | 1 | 1.12E-07 | RPS6 | Rapamycin |
| 1.09E-10 | 0.54755457 | 1 | 0.985 | 1.70E-06 | RPL37 | Rapamycin |
| 5.58E-10 | 0.51988984 | 1 | 1 | 8.71E-06 | RPLP2 | Rapamycin |
| 3.41E-09 | 0.6107096 | 1 | 0.857 | 5.32E-05 | RPL36 | Rapamycin |
| 3.69E-09 | -0.9354898 | 0.425 | 0.82 | 5.75E-05 | RP11-301G19.1 | Rapamycin |
| 6.34E-09 | 0.39064148 | 1 | 0.992 | 9.89E-05 | RPS19 | Rapamycin |
| 6.55E-09 | 0.53356793 | 1 | 0.985 | 0.00010212 | RPL37A | Rapamycin |
| 6.98E-09 | 0.47058689 | 1 | 0.992 | 0.00010891 | FAM211A | Rapamycin |
| 9.41E-09 | 0.67768655 | 1 | 1 | 0.00014686 | HBG2 | Rapamycin |
| 1.00E-08 | 0.43755456 | 1 | 0.992 | 0.00015652 | RPL12 | Rapamycin |
| 1.10E-08 | 0.46581519 | 1 | 0.985 | 0.00017218 | RPS21 | Rapamycin |
| 1.19E-08 | 0.39537124 | 1 | 1 | 0.00018541 | RPL31 | Rapamycin |
| 1.24E-08 | -0.5500528 | 0.95 | 0.985 | 0.00019338 | ENO1 | Rapamycin |
| 1.51E-08 | -0.6140742 | 0.975 | 0.992 | 0.0002362 | HSP90AA1 | Rapamycin |
| 2.52E-08 | 0.59391855 | 1 | 1 | 0.00039344 | HBG1 | Rapamycin |
| 4.38E-08 | -0.5155356 | 0.925 | 0.97 | 0.00068366 | NPM1 | Rapamycin |
| 4.52E-08 | -1.0412181 | 0.1 | 0.609 | 0.00070587 | GDF15 | Rapamycin |
| 5.48E-08 | 0.38646785 | 1 | 0.992 | 0.0008552 | COX7C | Rapamycin |
| 6.38E-08 | 0.55059372 | 1 | 0.887 | 0.00099493 | UQCRB | Rapamycin |
| 8.60E-08 | 0.78322717 | 0.875 | 0.692 | 0.00134174 | GAS5 | Rapamycin |
| 9.99E-08 | 0.39272896 | 1 | 1 | 0.00155842 | RPS14 | Rapamycin |
| 1.57E-07 | 0.27844811 | 1 | 0.992 | 0.00244998 | RPL30 | Rapamycin |
| 1.77E-07 | 0.41192472 | 1 | 1 | 0.00275386 | C17orf76-AS1 | Rapamycin |
| 2.49E-07 | -0.9352548 | 0.05 | 0.519 | 0.00388371 | PSAT1 | Rapamycin |
| 2.85E-07 | -0.7052082 | 0.55 | 0.842 | 0.00444492 | HSPA9 | Rapamycin |
| 2.91E-07 | -0.3995433 | 1 | 0.985 | 0.00454678 | GAPDH | Rapamycin |
| 3.88E-07 | 0.30481365 | 1 | 1 | 0.00604894 | RPS8 | Rapamycin |
| 5.44E-07 | 0.34859772 | 1 | 0.992 | 0.0084882 | RPL35A | Rapamycin |
| 7.76E-07 | -0.9133526 | 0.225 | 0.647 | 0.01210763 | ATF4 | Rapamycin |
| 7.81E-07 | 0.44318417 | 0.975 | 0.962 | 0.01218986 | ATP5E | Rapamycin |
| 1.04E-06 | -0.8524864 | 0.2 | 0.617 | 0.01618713 | FADS1 | Rapamycin |
| 1.18E-06 | -0.2861821 | 1 | 1 | 0.01841929 | FTL | Rapamycin |
| 1.55E-06 | 0.6631994 | 0.75 | 0.526 | 0.02411417 | NDUFB1 | Rapamycin |
| 2.32E-06 | -0.770062 | 0.35 | 0.752 | 0.03625128 | LYAR | Rapamycin |
| 2.60E-06 | -0.5897286 | 0.325 | 0.737 | 0.04057944 | SCD | Rapamycin |
| 2.64E-06 | -0.8995059 | 0.35 | 0.677 | 0.04118092 | MTHFD2 | Rapamycin |
| 2.81E-06 | 0.47978748 | 1 | 0.782 | 0.04378981 | EEF2 | Rapamycin |
| 2.97E-06 | -0.6623916 | 0.65 | 0.88 | 0.04630445 | EEF1E1 | Rapamycin |
| 2.99E-06 | 0.26542923 | 1 | 0.992 | 0.04661792 | RPL11 | Rapamycin |
| 3.19E-06 | -0.7392251 | 0.85 | 0.94 | 0.04970224 | NEAT1 | Rapamycin |
| 3.85E-21 | -2.6135151 | 0.195 | 1 | 6.01E-17 | HIST1H4C | Vinorelbine |
| 2.87E-18 | -1.0931994 | 0.951 | 1 | 4.48E-14 | TUBA1B | Vinorelbine |
| 1.57E-17 | -1.8288361 | 0.293 | 0.917 | 2.45E-13 | BEX4 | Vinorelbine |
| 1.70E-17 | 1.27593881 | 0.488 | 0 | 2.65E-13 | CLU | Vinorelbine |
| 5.40E-17 | -1.6844051 | 0.22 | 0.932 | 8.42E-13 | TUBB | Vinorelbine |
| 2.31E-16 | 1.34093271 | 0.976 | 0.872 | 3.60E-12 | ACTG1 | Vinorelbine |
| 4.06E-15 | 1.15779142 | 1 | 0.895 | 6.33E-11 | LGALS1 | Vinorelbine |
| 5.58E-13 | -1.2675846 | 0.268 | 0.91 | 8.70E-09 | TUBA1C | Vinorelbine |
| 6.22E-13 | -1.6660432 | 0.073 | 0.752 | 9.70E-09 | HIST1H1E | Vinorelbine |
| 6.82E-13 | -1.0345288 | 0.61 | 0.985 | 1.06E-08 | ALDOA | Vinorelbine |
| 1.06E-12 | -1.5064489 | 0.366 | 0.94 | 1.65E-08 | HIST1H1C | Vinorelbine |
| 1.86E-12 | -1.7168996 | 0.22 | 0.827 | 2.90E-08 | DDIT4 | Vinorelbine |
| 2.32E-12 | -1.3360705 | 0.171 | 0.812 | 3.61E-08 | HN1 | Vinorelbine |
| 5.13E-12 | 1.01488717 | 1 | 0.91 | 8.01E-08 | VIM | Vinorelbine |
| 9.08E-12 | -1.6023144 | 0 | 0.662 | 1.42E-07 | HIST1H1D | Vinorelbine |
| 1.43E-11 | -1.013949 | 0.463 | 0.902 | 2.24E-07 | BRK1 | Vinorelbine |
| 1.02E-10 | -0.6620726 | 0.927 | 0.985 | 1.58E-06 | GAPDH | Vinorelbine |
| 1.05E-10 | 0.62226865 | 1 | 0.992 | 1.64E-06 | RPS18 | Vinorelbine |
| 1.81E-10 | 1.18695627 | 0.659 | 0.211 | 2.82E-06 | CREM | Vinorelbine |
| 1.88E-10 | 0.80799816 | 0.976 | 0.97 | 2.94E-06 | TMSB10 | Vinorelbine |
| 3.50E-10 | 0.97069296 | 0.951 | 0.91 | 5.46E-06 | TMSB4X | Vinorelbine |
| 3.70E-10 | 0.4874192 | 1 | 0.977 | 5.78E-06 | RPL13A | Vinorelbine |
| 4.39E-10 | -0.6100379 | 0.854 | 0.932 | 6.84E-06 | PARK7 | Vinorelbine |
| 4.58E-10 | -1.2467763 | 0.146 | 0.737 | 7.14E-06 | CDK1 | Vinorelbine |
| 5.32E-10 | 0.89169131 | 0.976 | 0.895 | 8.29E-06 | S100A6 | Vinorelbine |
| 5.32E-10 | -0.8260484 | 0.902 | 1 | 8.30E-06 | MT-CO3 | Vinorelbine |
| 5.63E-10 | 0.438225 | 1 | 1 | 8.79E-06 | FTL | Vinorelbine |
| 6.18E-10 | -1.0091195 | 0.244 | 0.767 | 9.63E-06 | UBE2I | Vinorelbine |
| 6.36E-10 | 0.97664021 | 0.927 | 0.812 | 9.91E-06 | ACTB | Vinorelbine |
| 1.74E-09 | -0.8452248 | 0.683 | 0.917 | 2.72E-05 | NCL | Vinorelbine |
| 1.77E-09 | 1.11346801 | 1 | 1 | 2.76E-05 | RNA28S5 | Vinorelbine |
| 2.40E-09 | -0.7378039 | 0.732 | 0.947 | 3.75E-05 | YWHAB | Vinorelbine |
| 2.46E-09 | -0.9582325 | 0.39 | 0.805 | 3.84E-05 | FBL | Vinorelbine |
| 3.52E-09 | -0.8397028 | 0.707 | 0.962 | 5.49E-05 | CALM2 | Vinorelbine |
| 5.97E-09 | 1.01525365 | 0.439 | 0.075 | 9.31E-05 | PLAUR | Vinorelbine |
| 6.81E-09 | 1.10311109 | 0.976 | 0.91 | 0.00010622 | ZNF225 | Vinorelbine |
| 8.19E-09 | -1.0044677 | 0.415 | 0.797 | 0.00012773 | EPRS | Vinorelbine |
| 1.01E-08 | 0.9470963 | 0.878 | 0.692 | 0.00015786 | GAS5 | Vinorelbine |
| 1.06E-08 | 0.90802144 | 0.927 | 0.857 | 0.00016584 | RPS25 | Vinorelbine |
| 1.12E-08 | 0.94218284 | 0.415 | 0.068 | 0.0001741 | MT-TL1 | Vinorelbine |
| 1.12E-08 | 0.65278263 | 0.39 | 0.053 | 0.00017481 | S100A10 | Vinorelbine |
| 1.19E-08 | -0.7704057 | 0.537 | 0.91 | 0.00018628 | NME1 | Vinorelbine |
| 1.43E-08 | -0.8017896 | 0.39 | 0.82 | 0.00022268 | RP11-301G19.1 | Vinorelbine |
| 1.82E-08 | -0.9427646 | 0.537 | 0.842 | 0.00028394 | FDPS | Vinorelbine |
| 2.17E-08 | -0.7309242 | 0.366 | 0.895 | 0.00033836 | HBA2 | Vinorelbine |
| 2.98E-08 | -0.7204097 | 0.683 | 0.932 | 0.0004642 | NDUFAB1 | Vinorelbine |
| 3.03E-08 | -0.8851236 | 0.585 | 0.932 | 0.00047344 | HBA1 | Vinorelbine |
| 3.22E-08 | 0.91774422 | 0.805 | 0.504 | 0.00050252 | ANXA5 | Vinorelbine |
| 4.19E-08 | -0.952542 | 0.195 | 0.639 | 0.00065431 | DNPH1 | Vinorelbine |
| 4.45E-08 | 0.61493799 | 0.902 | 0.955 | 0.00069346 | MYL6 | Vinorelbine |
| 4.88E-08 | -0.6802752 | 0.512 | 0.925 | 0.00076176 | NMU | Vinorelbine |
| 5.18E-08 | -0.4162069 | 1 | 1 | 0.00080781 | SLC25A5 | Vinorelbine |
| 5.82E-08 | -0.822017 | 0.39 | 0.827 | 0.00090778 | NHP2L1 | Vinorelbine |
| 5.94E-08 | -0.7808272 | 0.439 | 0.812 | 0.00092615 | HNRNPDL | Vinorelbine |
| 6.65E-08 | -0.769473 | 0.366 | 0.789 | 0.0010368 | PDIA6 | Vinorelbine |
| 6.66E-08 | -0.8660319 | 0.439 | 0.842 | 0.00103821 | HSPA9 | Vinorelbine |
| 7.45E-08 | 0.80522013 | 0.902 | 0.774 | 0.0011616 | ANXA2 | Vinorelbine |
| 7.63E-08 | -0.9201962 | 0.146 | 0.609 | 0.00119104 | GPI | Vinorelbine |
| 9.27E-08 | -0.4221627 | 0.951 | 1 | 0.00144625 | RPL23 | Vinorelbine |
| 9.61E-08 | 0.68239154 | 0.878 | 0.767 | 0.00149894 | CLIC1 | Vinorelbine |
| 9.62E-08 | -0.8809665 | 0.098 | 0.586 | 0.0015011 | VPS25 | Vinorelbine |
| 9.76E-08 | -0.8233974 | 0.098 | 0.602 | 0.00152221 | CCDC12 | Vinorelbine |
| 1.08E-07 | -0.7401566 | 0.634 | 0.895 | 0.00169166 | MRPL14 | Vinorelbine |
| 1.13E-07 | -0.6554245 | 0.707 | 0.917 | 0.00176711 | ATP5B | Vinorelbine |
| 1.55E-07 | 0.60231328 | 0.927 | 0.812 | 0.00241457 | RPL3 | Vinorelbine |
| 1.57E-07 | -0.7871254 | 0.146 | 0.624 | 0.00244832 | HMBS | Vinorelbine |
| 1.60E-07 | -0.3297153 | 0.976 | 1 | 0.00248916 | RPS5 | Vinorelbine |
| 1.60E-07 | -0.8088394 | 0.049 | 0.511 | 0.00250269 | IDH1 | Vinorelbine |
| 1.66E-07 | -0.6346469 | 0.78 | 0.962 | 0.00259248 | BLVRB | Vinorelbine |
| 1.66E-07 | 1.03133743 | 0.756 | 0.549 | 0.00259429 | RHOC | Vinorelbine |
| 1.72E-07 | -0.5328344 | 0.61 | 0.865 | 0.00268754 | UROD | Vinorelbine |
| 1.79E-07 | -0.6702254 | 0.488 | 0.835 | 0.00278503 | APRT | Vinorelbine |
| 2.34E-07 | -0.4241635 | 0.976 | 1 | 0.00364702 | ATP5G3 | Vinorelbine |
| 2.38E-07 | -0.6616957 | 0.537 | 0.895 | 0.00371873 | TCEAL8 | Vinorelbine |
| 2.66E-07 | -0.8978028 | 0.049 | 0.519 | 0.00414505 | PSAT1 | Vinorelbine |
| 2.67E-07 | -0.8479107 | 0.22 | 0.684 | 0.0041709 | ID1 | Vinorelbine |
| 2.71E-07 | -0.9221675 | 0.024 | 0.489 | 0.00422662 | FAM178B | Vinorelbine |
| 3.26E-07 | 0.96511146 | 0.341 | 0.053 | 0.00508117 | LGALS3 | Vinorelbine |
| 3.52E-07 | -1.0146446 | 0.463 | 0.82 | 0.00548679 | TOP2A | Vinorelbine |
| 3.68E-07 | -0.7057051 | 0.268 | 0.737 | 0.00574502 | WBP5 | Vinorelbine |
| 3.74E-07 | -0.7416765 | 0.902 | 0.97 | 0.00583128 | MT-ND4 | Vinorelbine |
| 3.79E-07 | 1.07233391 | 0.829 | 0.624 | 0.00590501 | ANXA1 | Vinorelbine |
| 4.65E-07 | 0.64615237 | 0.293 | 0.03 | 0.00724877 | IFI16 | Vinorelbine |
| 5.56E-07 | -0.8239789 | 0.22 | 0.647 | 0.00867076 | EIF4EBP1 | Vinorelbine |
| 6.23E-07 | -0.7563751 | 0.366 | 0.797 | 0.00971291 | UTP11L | Vinorelbine |
| 6.29E-07 | 0.86525934 | 0.854 | 0.617 | 0.00981827 | CCNB1 | Vinorelbine |
| 6.51E-07 | -0.6958311 | 0.268 | 0.737 | 0.01015772 | SCD | Vinorelbine |
| 6.80E-07 | 0.8115246 | 0.707 | 0.316 | 0.01060569 | ARAP1 | Vinorelbine |
| 8.11E-07 | 0.3776463 | 1 | 0.992 | 0.01265426 | RPS19 | Vinorelbine |
| 8.28E-07 | -0.4978643 | 0.854 | 0.94 | 0.01291884 | LDHB | Vinorelbine |
| 9.02E-07 | -0.6781625 | 0.561 | 0.865 | 0.0140718 | STRA13 | Vinorelbine |
| 9.07E-07 | -0.6515304 | 0.537 | 0.85 | 0.01415579 | RBM3 | Vinorelbine |
| 9.98E-07 | -0.7375722 | 0.341 | 0.737 | 0.01556404 | SARS | Vinorelbine |
| 1.01E-06 | 0.60977426 | 0.902 | 0.895 | 0.0157899 | CKS2 | Vinorelbine |
| 1.15E-06 | 0.81674142 | 0.39 | 0.09 | 0.01793171 | CNN1 | Vinorelbine |
| 1.19E-06 | -0.784793 | 0.049 | 0.489 | 0.01855349 | FBXO5 | Vinorelbine |
| 1.32E-06 | -0.8533543 | 0.244 | 0.677 | 0.02057125 | MTHFD2 | Vinorelbine |
| 1.39E-06 | -0.6211903 | 0.049 | 0.474 | 0.02167602 | FARSA | Vinorelbine |
| 1.40E-06 | 0.96964957 | 0.805 | 0.617 | 0.02178548 | TIMP1 | Vinorelbine |
| 1.71E-06 | -0.6169142 | 0.073 | 0.489 | 0.02671478 | SHMT2 | Vinorelbine |
| 1.79E-06 | 0.82659482 | 0.854 | 0.624 | 0.02799057 | HMMR | Vinorelbine |
| 1.82E-06 | -0.7330714 | 0.439 | 0.812 | 0.02845551 | PRDX2 | Vinorelbine |
| 1.96E-06 | -0.4704874 | 0.585 | 0.872 | 0.03062855 | ATP5A1 | Vinorelbine |
| 2.25E-06 | 0.62446372 | 0.341 | 0.068 | 0.03513483 | SMIM14 | Vinorelbine |
| 2.44E-06 | 0.27458955 | 1 | 1 | 0.03811678 | RPLP1 | Vinorelbine |
| 2.97E-06 | -0.6520561 | 0.098 | 0.511 | 0.0463965 | CYBA | Vinorelbine |
| 3.06E-06 | -0.6255555 | 0.146 | 0.586 | 0.04780546 | CTSH | Vinorelbine |

**Supplementary Table 4.** cDNA concentration obtained according to the amount of beads per PCR mix.

| **Bead amount per PCR mix** | **Control** | **80 µg** | **100 µg** | **120 µg** | **140 µg** | **160 µg** | **180 µg** | **200 µg** |
| --- | --- | --- | --- | --- | --- | --- | --- | --- |
|  | **(0 µg)** |  |  |  |  |  |  |  |
| **Conc. (ng/µL)** | > 50 | 23.1 | 10.9 | 5.79 | 4.4 | 3.82 | 3.01 | 2.5 |

**Supplementary Table 5.** Cost analysis of the MAPS-seq method.

| Product | Cat. # | Supplier | Cost for 96 cells ($) | Cost for 384 cells ($) |
| --- | --- | --- | --- | --- |
| Streptavidin C1 beads | 65001 | Invitrogen | 18.67 | 74.69 |
| Maxima H– Reverse Transcriptase | EP0753 | Thermo Scientific | 6.48 | 6.48 |
| dNTP mix | N0447L | NEB | 0.25 | 0.25 |
| RNase inhibitor | Y9240L | Enzymatics | 0.74 | 0.74 |
| Exonuclease I | 0293L | NEB | 3.68 | 3.68 |
| KAPA Hifi HotStart ReadyMix | KK2602 | KAPA Biosystems | 10.94 | 43.76 |
| Nextera XT DNA sample preparation kit | FC-131-1024 | Illumina | 45.83 | 45.83 |
| AMPure XP beads | A63881 | Beckman Coulter | 1.66 | 1.66 |
| Total cost | | | 88.25 | 177.09 |
| Cost per cell | | | 0.92 | 0.46 |
